# Genetic Predictive Factors for Nonsusceptible Phenotypes and Multidrug Resistance in Expanded-Spectrum Cephalosporin-Resistant Uropathogenic Escherichia coli from a Multicenter Cohort: Insights into the Phenotypic and Genetic Basis of Coresistance

**DOI:** 10.1128/msphere.00471-22

**Published:** 2022-11-15

**Authors:** Nicole Jackson, Cheyenne R. Belmont, Nicole J. Tarlton, Yuan Hu Allegretti, Sheila Adams-Sapper, Yolanda Yue Huang, Clarissa A. Borges, Bradley W. Frazee, Danka Florence-Petrovic, Clarisse Hufana, Anna Parker, Claire F. Mastrangelo, Shevya Awasthi, Isha Kane, Zlatan Coralic, Steve Miller, Joycelyn Diaz, Christopher Fee, Cassiana E. Bittencourt, Omai Garner, Sukantha Chandrasekaran, Claudia Crandall, Julian C. Marcha, Mir H. Noorbakhsh, Patricia Rodrigues-Wong, Tara R. deBoer, Lee W. Riley

**Affiliations:** a School of Public Health, Division of Infectious Diseases and Vaccinology, University of California, Berkeley, California, USA; b School of Public Health, Division of Epidemiology, University of California, Berkeley, California, USA; c Department of Microbiology, BioAmp Diagnostics, Inc., San Carlos, California, USA; d Department of Chemistry, BioAmp Diagnostics, Inc., San Carlos, California, USA; e Division of Environmental Genomics and Systems Biology, Lawrence Berkeley National Laboratory, Berkeley, California, USA; f Department of Emergency Medicine, Highland Hospital, Alameda Health System, Oakland, California, USA; g Department of Laboratory Medicine and Pathology, Highland Hospital, Alameda Health System, Oakland, California, USA; h Department of Pharmacy, University of California, San Francisco, California, USA; i Department of Laboratory Medicine, University of California, San Francisco, California, USA; j Department of Pathology and Laboratory Medicine, University of California, Irvine, California, USA; k Department of Pathology and Laboratory Medicine, University of California, Los Angeles, California, USA; l John Muir Microbiology, John Muir Health, Concord, California, USA; m Sutter Shared Lab, Sutter Health, Livermore, California, USA; n Department of Emergency Medicine, University of California, San Francisco, California, USA; CDC

**Keywords:** ESBL, expanded-spectrum cephalosporin resistance, multilocus sequence type, uropathogenic *E. coli*, whole-genome sequence

## Abstract

Antimicrobial resistance in urinary tract infections (UTIs) is a major public health concern. This study aims to characterize the phenotypic and genetic basis of multidrug resistance (MDR) among expanded-spectrum cephalosporin-resistant (ESCR) uropathogenic Escherichia coli (UPEC) causing UTIs in California patient populations. Between February and October 2019, 577 ESCR UPEC isolates were collected from patients at 6 clinical laboratory sites across California. Lineage and antibiotic resistance genes were determined by analysis of whole-genome sequence data. The lineages ST131, ST1193, ST648, and ST69 were predominant, representing 46%, 5.5%, 4.5%, and 4.5% of the collection, respectively. Overall, 527 (91%) isolates had an expanded-spectrum β-lactamase (ESBL) phenotype, with *bla*_CTX-M-15_, *bla*_CTX-M-27_, *bla*_CTX-M-55_, and *bla*_CTX-M-14_ being the most prevalent ESBL genes. In the 50 non-ESBL phenotype isolates, 40 (62%) contained *bla*_CMY-2_, which was the predominant plasmid-mediated AmpC (pAmpC) gene. Narrow-spectrum β-lactamases, *bla*_TEM-1B_ and *bla*_OXA-1_, were also found in 44.9% and 32.1% of isolates, respectively. Among ESCR UPEC isolates, isolates with an ESBL phenotype had a 1.7-times-greater likelihood of being MDR than non-ESBL phenotype isolates (*P* < 0.001). The cooccurrence of *bla*_CTX-M-15_, *bla*_OXA-1_, and *aac(6*′*)-Ib-cr* within ESCR UPEC isolates was strongly correlated. Cooccurrence of *bla*_CTX-M-15_, *bla*_OXA-1_, and *aac(6*′*)-Ib-cr* was associated with an increased risk of nonsusceptibility to piperacillin-tazobactam, cefepime, fluoroquinolones, and amikacin as well as MDR. Multivariate regression revealed the presence of *bla*_CTX-M-55_, *bla*_TEM-1B_, and the ST131 genotype as predictors of MDR.

**IMPORTANCE** The rising incidence of resistance to expanded-spectrum cephalosporins among Escherichia coli strains, the most common cause of UTIs, is threatening our ability to successfully empirically treat these infections. ESCR E. coli strains are often MDR; therefore, UTI caused by these organisms often leads to treatment failure, increased length of hospital stay, and severe complications (D. G. Mark, Y.-Y. Hung, Z. Salim, N. J. Tarlton, et al., Ann Emerg Med 78:357–369, 2021, https://doi.org/10.1016/j.annemergmed.2021.01.003). Here, we performed an in-depth analysis of genetic factors of ESCR E. coli associated with coresistance and MDR. Such knowledge is critical to advance UTI diagnosis, treatment, and antibiotic stewardship.

## INTRODUCTION

Urinary tract infections (UTIs) are among the most common bacterial infections in both community and hospital settings, accounting for >10 million ambulatory visits and incurring $1.6 to 2.8 billion in health care costs annually in the United States alone (1–4). Moreover, UTIs are the third most common reason for oral antibiotic prescriptions (5). The rising prevalence of multidrug-resistant (MDR) uropathogens increasingly impacts the management of UTIs, with expanded-spectrum cephalosporin-resistant *Enterobacterales* (ESCR-E) causing particular concern (6, 7). The reported prevalence of these organisms among community- and hospital-onset UTI isolates is now 15 to 17% (8). ESCR-E have been classified as a serious threat to public health and a critical priority for new antimicrobial development by the Centers for Disease Control and Prevention and the World Health Organization (9, 10). Phenotypic resistance to expanded-spectrum cephalosporins (ESC; third-generation cephalosporins) is conferred predominantly by extended-spectrum β-lactamases (ESBLs) and chromosomal or plasmid-mediated AmpC (cAmpC or pAmpC, respectively) β-lactamases. ESBLs hydrolyze penicillins, oxyimino-cephalosporins (ceftazidime, cefotaxime, ceftriaxone, and cefepime), and monobactams (aztreonam), whereas cAmpC and pAmpC enzymes possess a similar spectrum of activity, though they do not hydrolyze cefepime (11, 12).

Major UTI syndromes include cystitis, pyelonephritis, and prostatitis, with complicated UTIs (cUTIs) being defined as those that occur in the setting of immunosuppression or urinary obstruction, carrying a higher risk of treatment failure (13–15). Uncomplicated cystitis is generally treated empirically with oral antibiotics, without obtaining urine culture (16). However, urine culture is strongly recommended in cases of pyelonephritis and cUTI (17). Initial broad-spectrum empirical treatment, typically with an oral fluoroquinolone or parenteral third-generation cephalosporin, can potentially be stepped down based on culture and susceptibility results (14). Empirical treatment that is discordant with susceptibility results occurs more frequently in ESCR than ESC-susceptible UTI and is associated with prolonged hospitalization and higher mortality (6, 18–20). Treatment of ESCR-E infections is further complicated by the high rates of coresistance to non-β-lactam antimicrobial agents (fluoroquinolones, trimethoprim-sulfamethoxazole, and aminoglycosides), which limit treatment options and promote the use of broader-spectrum agents (12, 21). Consequently, carbapenems are increasingly used for both empirical treatment of cUTI and culture-directed treatment of ESCR-UTI (22, 23). This trend is concerning, as carbapenems are considered “last-line” therapy for Gram-negative bacterial infections, and increased use is associated with the emergence of carbapenem-resistant Enterobacterales (CRE) (24–26). To preserve the effectiveness of last-line antibiotics, there have been calls to develop and evaluate carbapenem-sparing strategies in ESCR-UTI management (23, 27, 28).

Although ESCR-E have been broadly investigated worldwide, there have been no large-scale multicenter genomic studies of uropathogenic ESCR-E. The overarching aim of this study is to understand the phenotypic and genetic basis of coresistance within ESCR uropathogenic Escherichia coli (UPEC), focusing on the genetic predictors of coresistance and MDR. This study focused on UPEC, the most common causative pathogen in UTIs, accounting for roughly 70% of all cases (29). Increasing our understanding of genetic predictors for MDR and coresistance within ESCR UPEC strains could provide opportunities for improved surveillance and prescribing practices for resistant uropathogens.

## RESULTS

### Patient demographic characteristics, clinical specimens, and isolate typing information.

Five hundred seventy-seven E. coli isolates, resistant to at least one third-generation cephalosporin (ESCR UPEC), were included in this analysis. The source of urine specimens included voided urine (*n* = 269, 46.6%) and bladder catheterization (*n* = 84, 14.6%) or was unknown (Table 1). Among the study population, 409 (70.9%) of 577 samples originated from females, and patient age ranged from 0 to 102 years, with the age group 64 to 79 years contributing 173 (30%) samples. Samples were obtained from 6 clinical laboratory sites across California; site 4 contributed 145 (25.1%) isolates, while the remaining 5 sites contributed 81 to 106 isolates each. Overall, 527 (91.3%) isolates were phenotypically confirmed to produce an ESBL (positive by ESBL confirmatory testing according to CLSI). Male sex was associated with MDR (*P* = 0.0024). Prevalence of ESBL-producing UPEC ranged from 12.9% to 26% across locations (*P* = 0.004). We did not have access to the type of UTI syndrome associated with each specimen or the clinical information needed to determine whether the UTI was considered a complicated infection.

**TABLE 1:** Patient characteristics and distribution of ESCR UPEC isolates by site, stratified by MDR and ESBL phenotype^*a*^

Patient characteristic	No. (%) of isolates by phenotype:				
	Non-MDR (*n* = 189)	MDR (*n* = 388)	Non-ESBL (*n* = 50)	ESBL (*n* = 527)	Overall (*n* = 577)
Sex (male)	43 (22.8)	125 (32.2)	10 (20)	158 (30)	168 (29.1)
Age (yr)					
0–17	6 (3.2)	10 (2.6)	2 (4.0)	14 (2.7)	16 (2.8)
18–44	46 (24.3)	59 (15.2)	14 (28.0)	91 (17.3)	105 (18.2)
45–63	51 (27.0)	109 (28.1)	8 (16.0)	152 (28.8)	160 (27.7)
64–79	55 (29.1)	118 (30.4)	16 (32.0)	157 (29.8)	173 (30.0)
80+	31 (16.4)	92 (23.7)	10 (20.0)	113 (21.4)	123 (21.3)
Specimen					
Catheter	26 (13.8)	58 (14.9)	5 (10.0)	79 (15.0)	84 (14.6)
Voided	93 (49.2)	176 (45.4)	33 (66.0)	236 (44.8)	269 (46.6)
Surgical	3 (1.6)	6 (1.5)	1 (2.0)	8 (1.5)	9 (1.6)
Suprapubic aspirate	1 (0.5)	1 (0.3)	0 (0)	2 (0.4)	2 (0.3)
Unknown	66 (34.9)	147 (37.9)	11 (22.0)	202 (38.3)	213 (36.9)
Location					
Site 1	27 (14.3)	53 (13.7)	12 (24)	68 (12.9)	80 (13.9)
Site 2	26 (13.8)	64 (16.5)	13 (26)	77 (14.6)	90 (15.6)
Site 3	20 (10.6)	61 (15.7)	1 (2)	80 (15.2)	81 (14)
Site 4	48 (25.4)	97 (25)	8 (16)	137 (26)	145 (25.1)
Site 5	29 (15.3)	46 (11.9)	4 (8)	71 (13.5)	75 (13)
Site 6	39 (21)	67 (17.3)	12 (24)	94 (17.8)	106 (8.4)

a An MDR isolate was defined by phenotypic resistance to at least 1 agent in ≥3 classes of antimicrobial agents used to treat UTI (β-lactams, fluoroquinolones, aminoglycosides, trimethoprim-sulfamethoxazole, and nitrofurantoin). An ESBL phenotype was defined by confirmation of ESBL production by the CLSI disk diffusion ESBL confirmatory method.

From genotyping the 577 ESCR UPEC isolates, 7 of the 8 known E. coli phylogenetic groups were identified, which included 482 (83.5%) isolates from the extraintestinal pathogenic E. coli (ExPEC)-associated phylogroups—356 (61.7%) from B2, 75 (13%) from D, and 51 (8.8%) from A, in addition to 95 (16.5%) isolates from other phylogroups (see Fig. S1 in the supplemental material). Further characterization of the collection by multilocus sequence typing (MLST) revealed 73 distinct sequence types, as well as 4 isolates of unknown MLST. The predominant MLSTs were ST131 (46%), ST1193 (5.5%), ST648 (4.5%), ST69 (4.5%), ST38 (3.8%), ST636 (3.1%), and ST410 (1.7%).

10.1128/msphere.00471-22.1 FIG S1 (A) Proportions of phylogroups identified. (B) Pie plot displaying prevalence of MLSTs, sorted by clinical laboratory site (sites1 to 6). Diagrams were created using ggplot2 in R 3.0.1. Download FIG S1, DOCX file, 0.5 MB. Copyright © 2022 Jackson et al. 2022 Jackson et al. https://creativecommons.org/licenses/by/4.0/ This content is distributed under the terms of the Creative Commons Attribution 4.0 International license.

### Antimicrobial susceptibility and multidrug resistance profiles in ESBL and non-ESBL phenotype ESCR UPEC.

**FIG 1:**
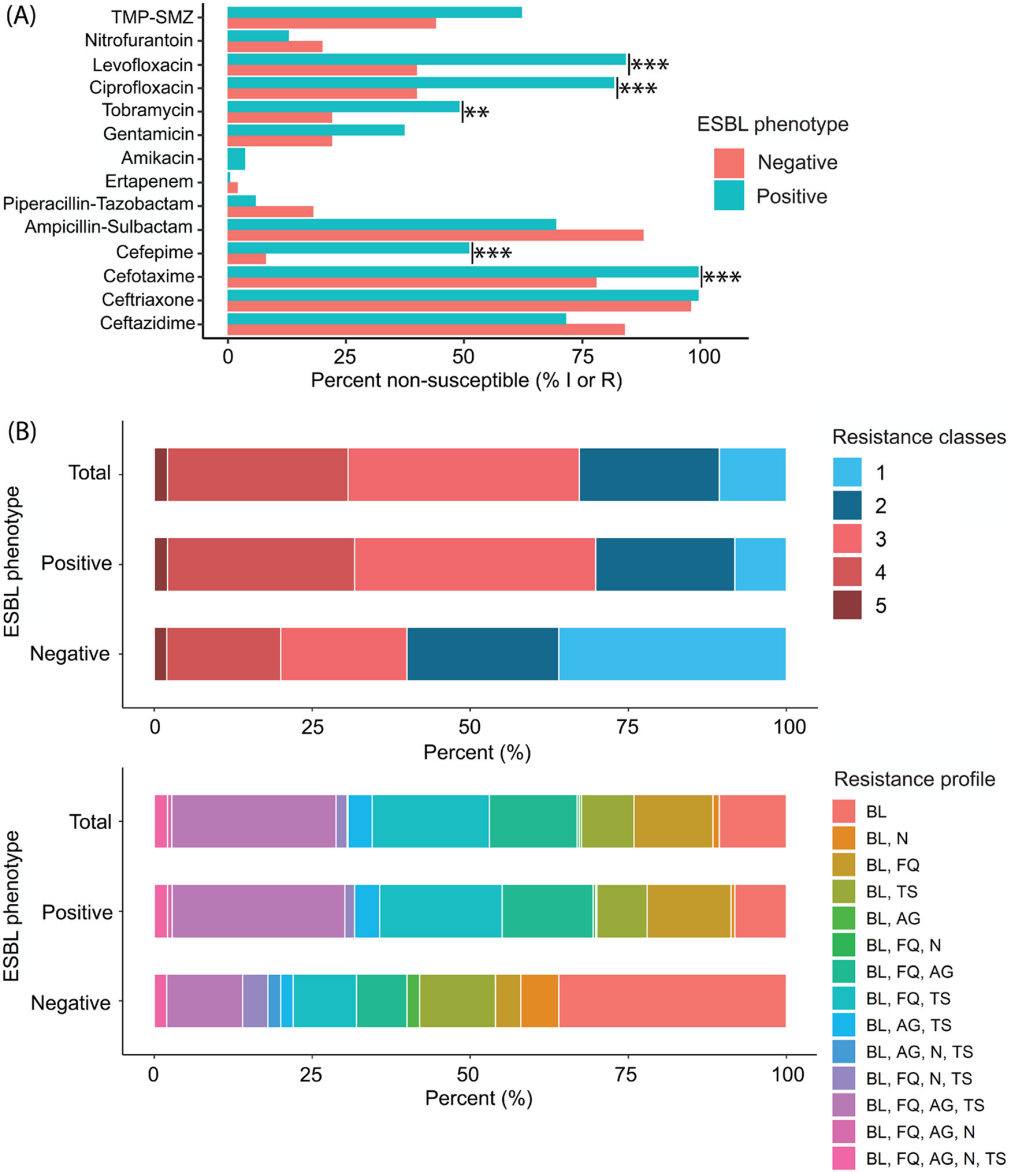
Antimicrobial resistance and MDR profiles in ESCR UPEC isolates (*n* = 577), stratified by ESBL phenotype. (A) Antimicrobial nonsusceptibility of ESBL and non-ESBL phenotype ESCR UPEC isolates. Isolates interpreted as intermediate or resistant (I or R, respectively) according to CLSI breakpoints (66) were categorized as “nonsusceptible.” (B) Stacked bar charts displaying prevalence of MDR and characterization of resistance profiles among ESBL and non-ESBL phenotype ESCR UPEC isolates. “Resistance classes” describes the number of distinct antimicrobial classes that isolates were resistant to. Abbreviations: BL, β-lactams; N, nitrofurantoin; FQ, fluoroquinolones; AG, aminoglycosides; TMP-SMZ (A) or TS (B), trimethoprim-sulfamethoxazole. Statistical analyses were performed using Fisher’s exact test, and results were plotted using the ggplot2 function in R 3.0.1. Susceptibility data for all ESCR UPEC isolates (*n* = 577) were included in this analysis. *P* value: <0.05 (*), <0.01 (**), and <0.001 (***).

Antimicrobial susceptibility and coresistance of isolates with ESBL and non-ESBL phenotypes were characterized (Table 2 and Fig. 1). The frequency of antimicrobial nonsusceptibility differed between ESBL phenotype and non-ESBL phenotype ESCR UPEC isolates, including nonsusceptibility to ciprofloxacin (82% versus 40%, respectively; *P* < 0.001), levofloxacin (84% versus 40%, respectively; *P* < 0.001), tobramycin (49% versus 22%, respectively; *P* = 0.004), cefepime (51% versus 8%, respectively; *P* < 0.001), and cefotaxime (99.6% versus 78%, respectively; *P* < 0.001) (Fig. 1 and Table S1). Antimicrobial nonsusceptibility, ESBL phenotype, and MDR stratified by common MLSTs are shown in Table S2.

**TABLE 2:** ESCR UPEC susceptibility, stratified by ESBL phenotype^*a*^

Antimicrobial agent	No. (%) of isolates by phenotype and susceptibility								
	Non-ESBL (*n* = 50)			ESBL (*n* = 527)			Total (*n* = 577)		
	Susceptible	Intermediate	Resistant	Susceptible	Intermediate	Resistant	Susceptible	Intermediate	Resistant
β-Lactams or β-lactam–β-lactamase inhibitor combinations									
Ceftazidime	8 (16.0)	15 (30.0)	27 (54.0)	150 (28.5)	132 (25.0)	245 (46.5)	158 (27.4)	147 (25.5)	272 (47.1)
Ceftriaxone	1 (2.0)	4 (8.0)	45 (90.0)	2 (0.4)	0 (0)	525 (99.6)	3 (0.5)	4 (0.7)	570 (98.8)
Cefotaxime	11 (22.0)	3 (6.0)	36 (72.0)	2 (0.4)	3 (0.6)	522 (99.1)	13 (2.3)	6 (1.0)	558 (96.7)
Cefepime^*b*^	41 (82.0)	5 (10.0)^*b*^	4 (8.0)	187 (35.5)	71 (13.5)	269 (51.0)	228 (39.5)	76 (13.2)	273 (47.3)
Ampicillin-sulbactam	6 (12.0)	13 (26.0)	31 (62.0)	161 (30.6)	140 (26.6)	226 (42.9)	167 (28.9)	153 (26.5)	257 (44.5)
Piperacillin-tazobactam	41 (82.0)	5 (10.0)	4 (8.0)	496 (94.1)	19 (3.6)	12 (2.3)	537 (93.1)	24 (4.2)	16 (2.8)
Ertapenem	49 (98.0)	1 (2.0)	0 (0)	525 (99.6)	0 (0)	2 (0.4)	574 (99.5)	1 (0.2)	2 (0.3)
Aminoglycosides									
Amikacin	50 (100)	0 (0)	0 (0)	508 (96.4)	12 (2.3)	7 (1.3)	558 (96.7)	12 (2.1)	7 (1.2)
Gentamicin	39 (78.0)	0 (0)	11 (22.0)	330 (62.6)	4 (0.8)	193 (36.6)	369 (64.0)	4 (0.7)	204 (35.4)
Tobramycin	39 (78.0)	5 (10.0)	6 (12.0)	269 (51.0)	65 (12.3)	193 (36.6)	308 (53.4)	70 (12.1)	199 (34.5)
Fluoroquinolones									
Ciprofloxacin	30 (60.0)	0 (0)	20 (40.0)	96 (18.2)	14 (2.7)	417 (79.1)	126 (21.8)	14 (2.4)	437 (75.7)
Levofloxacin	30 (60.0)	0 (0)	20 (40.0)	83 (15.7)	46 (8.7)	398 (75.5)	113 (19.6)	46 (8.0)	418 (72.4)
Other									
Trimethoprim-sulfamethoxazole	28 (56.0)	0 (0)	22 (44.0)	199 (37.8)	0 (0)	328 (62.2)	227 (39.3)	0 (0)	350 (60.7)
Nitrofurantoin	40 (80.0)	1 (2.0)	9 (18.0)	459 (87.1)	40 (7.6)	28 (5.3)	499 (86.5)	41 (7.1)	37 (6.4)

a Phenotypic ESBL status and susceptibilities were determined in accordance with CLSI standards (53).

b In 2016 the intermediate breakpoint for cefepime was changed to susceptible dose-dependent (SDD), as isolates may remain susceptible to cefepime if the drug dose or frequency of administration is increased. As treatment of SDD organisms relies on accurate susceptibility/MIC determination (and therefore does not impact empirical therapies), for the purpose of this study we treated SDD isolates as intermediate or “nonsusceptible.”

10.1128/msphere.00471-22.2 TABLE S1 Antimicrobial nonsusceptibility (susceptibility categorized as intermediate or resistant in relation to CLSI breakpoints), stratified by phenotypic ESBL status. Statistical analyses were performed using Fisher’s exact test in R 3.0.1. Download Table S1, DOCX file, 0.01 MB. Copyright © 2022 Jackson et al. 2022 Jackson et al. https://creativecommons.org/licenses/by/4.0/ This content is distributed under the terms of the Creative Commons Attribution 4.0 International license.

10.1128/msphere.00471-22.3 TABLE S2 Antimicrobial nonsusceptibility (susceptibility categorized as intermediate or resistant in relation to CLSI breakpoints), stratified by the 6 most common MLST types identified in this collection. Download Table S2, DOCX file, 0.01 MB. Copyright © 2022 Jackson et al. 2022 Jackson et al. https://creativecommons.org/licenses/by/4.0/ This content is distributed under the terms of the Creative Commons Attribution 4.0 International license.

Overall, 388 (67%) isolates were MDR (Fig. 1). MDR prevalence was 1.7 times greater in ESBL phenotype than in non-ESBL phenotype UPEC isolates (69.8% versus 40%, respectively; *P* < 0.001) (Table S1). MDR prevalence also varied across predominant MLSTs, ranging from 0% in ST636 to 81.6% in ST131 (Table S2). We further characterized the resistance profiles of MDR isolates. Resistance to fluoroquinolones, aminoglycosides, and trimethoprim-sulfamethoxazole, in addition to β-lactams, was found in 150 (39%) isolates classified as MDR. Antimicrobial resistance profiles stratified by ESBL phenotype are shown in Table S3.

10.1128/msphere.00471-22.4 TABLE S3 Antimicrobial resistance profiles, stratified by phenotypic ESBL status. Statistical analyses were performed using Fisher’s exact test in R 3.0.1. Abbreviations: BL, β-lactam; N, nitrofurantoin; FQ, fluoroquinolones; AG, aminoglycosides; TS, trimethoprim-sulfamethoxazole. Download Table S3, DOCX file, 0.01 MB. Copyright © 2022 Jackson et al. 2022 Jackson et al. https://creativecommons.org/licenses/by/4.0/ This content is distributed under the terms of the Creative Commons Attribution 4.0 International license.

### Whole-genome sequence (WGS) analysis for identification of resistance mechanisms and replicon types in ESCR UPEC.

β-Lactamase genes, horizontally acquired resistance genes, and mutations known to confer antimicrobial resistance were characterized within the 577 ESCR UPEC isolates. Genes encoding β-lactamases known to confer resistance to ESC were identified in 566 (98%) of the ESCR UPEC isolates. *bla*_CTX-M_ ESBL genes were present in 527 (91.3%) isolates, whereas *bla*_CMY_ pAmpC genes were present in 48 (8.3%) isolates (Table S4). The carbapenemase variant *bla*_KPC-2_ was detected in one isolate. Narrow-spectrum (non-ESBL) *bla*_TEM_ genes were detected in 207 (44.9%) isolates, whereas narrow-spectrum (non-ESBL) *bla*_OXA_ genes were identified in 184 (32%) isolates. In 173 (29.9%) isolates, both *bla*_OXA-1_ and *bla*_CTX-M-15_ genes were identified. The prevalence of certain β-lactamase genes differed between ESCR UPEC isolates with ESBL and non-ESBL phenotypes, including variants of the *bla*_CTX-M_ (98.9% versus 12%, respectively; *P* < 0.001), *bla*_CMY_ (2.9% versus 66%, respectively; *P* < 0.001), and *bla*_OXA_ (34.4% versus 8.0%, respectively; *P* = 0.004) (Table S4).

10.1128/msphere.00471-22.5 TABLE S4 Isolates containing β-lactamase genes identified from the WGS analysis, stratified by ESBL phenotype. Statistical analyses were performed using Fisher’s exact test in R 3.0.1. Download Table S4, DOCX file, 0.02 MB. Copyright © 2022 Jackson et al. 2022 Jackson et al. https://creativecommons.org/licenses/by/4.0/ This content is distributed under the terms of the Creative Commons Attribution 4.0 International license.

Overall, 523 (99.6%) ESBL phenotype isolates versus 7 (14.0%) non-ESBL phenotype isolates carried a characterized ESBL gene, while 17 (3.2%) ESBL phenotype isolates versus 31 (62.0%) non-ESBL phenotype isolates carried a pAmpC gene (Table S4). Fourteen (2.3%) ESCR UPEC isolates contained both ESBL and pAmpC β-lactamase genes; of these, 13 (92.8%) had an ESBL phenotype whereas 1 (7.1%) had a non-ESBL phenotype. Eleven (1.9%) ESCR UPEC isolates contained no characterized β-lactamase genes which confer resistance to third-generation cephalosporins; within these isolates, 5 (38.5%) contained cAmpC promoter mutations, 4 (30.8%) contained a *bla*_TEM-1B_ gene, 1 (7.7%) contained a *bla*_OXA-1_ gene, 1 (7.7%) contained a *bla*_CARB-2_ gene, and 5 (45.5%) contained no characterized β-lactamase genes.

**FIG 2:**
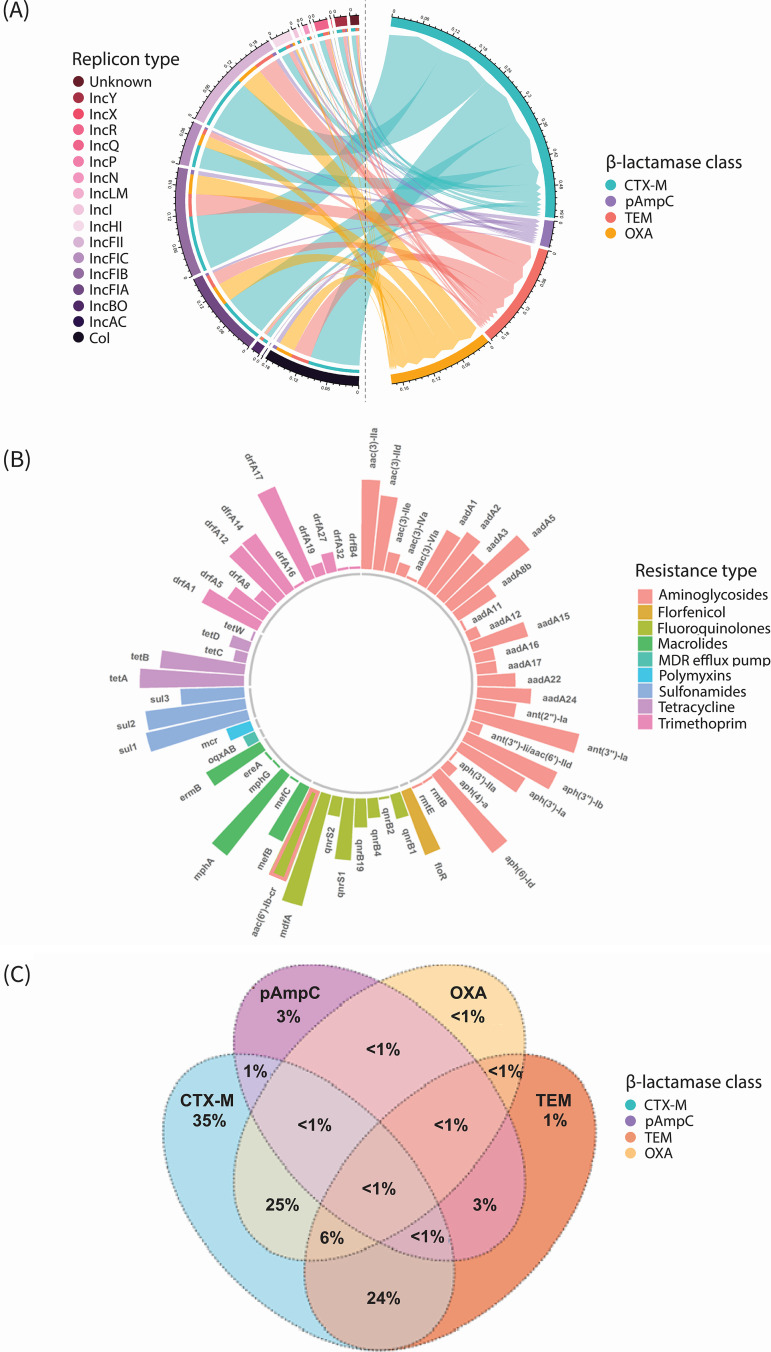
Replicon types, β-lactamase genes, and other horizontally acquired resistance determinants in ESCR UPEC isolates (*n* = 577), identified from WGS analysis. (A) Chord diagram displaying the replicon types and their association with β-lactamase classes. The chord plot displays replicon types/β-lactamase genes as a proportion of all replicon-gene combinations. (B) Sunburst plot displaying proportions (presented on a logarithmic scale) of horizontally acquired resistance mechanisms which provide protection against agents other than β-lactams. The bar representing the gene *aac(6*′*)-Ib-cr* is labeled both peach and light green, as this determinant confers resistance to both fluoroquinolones and aminoglycosides. (C) Venn diagram displaying relationships between prevalent β-lactamase gene classes and the percentage of isolates in which each gene class was identified. Diagrams were created with the chordDiagram, ggplot2, and VennDiagram packages using R 3.0.1.

Horizontally acquired genes which confer resistance to 8 distinct antimicrobial classes (other than β-lactams) were identified, including genes known to provide protection against agents commonly used to treat UTIs (trimethoprim-sulfamethoxazole, fluoroquinolones, and aminoglycosides) and genes conferring resistance to polymyxins, tetracyclines, macrolides, and florfenicol (Fig. 2). Several resistance genes differed in prevalence between ESBL and non-ESBL phenotype UPEC isolates: *aac(6*′*)-Ib-cr* (34.5% versus 8.0%, respectively; *P* = 0.004) and *aac(3)*-*IIa* (26.0% versus 4.0%, respectively; *P* = 0.026), which confer resistance to aminoglycosides (Table S5).

10.1128/msphere.00471-22.6 TABLE S5 Horizontally acquired resistance genes and mutations conferring antibiotic resistance, identified from WGS analysis and stratified by ESBL phenotype. Statistical analyses were performed using Fisher’s exact test in R 3.0.1. Download Table S5, DOCX file, 0.02 MB. Copyright © 2022 Jackson et al. 2022 Jackson et al. https://creativecommons.org/licenses/by/4.0/ This content is distributed under the terms of the Creative Commons Attribution 4.0 International license.

Mutations which are known to confer resistance to fluoroquinolones, polymyxins, and tetracyclines were also identified (Table S4). Regarding fluoroquinolone resistance, mutations in the gene encoding DNA gyrase subunit A were identified in 459 (79.5%) isolates, whereas mutations in topoisomerase IV genes were detected in 424 (73.5%) isolates. The proportion of DNA gyrase mutations *gyrA* (S83L) (83.7% versus 36%, *P* < 0.001) and *gyrA* (D87N) (68.5% versus 32%, *P* < 0.001) and the topoisomerase mutations *parC* (S80I) (72.5% versus 34.0%, *P *< 0.001), *parC* (E84V) (43.5% versus 10.0%, *P* < 0.001), and *parE* (I529L) (46.9% versus 12.0%, *P* < 0.001) differed between ESBL phenotype and non-ESBL phenotype isolates, respectively (Table S5). Regarding polymyxin resistance, a *pmrA* gene mutation (R81S) was identified in 1 (0.2%) isolate. A mutation in the 16S rRNA operon *rrsB* (G1058C), conferring resistance to tetracyclines, was also identified in 1 isolate (0.2%) (Table S5).

Regarding plasmid content, 16 distinct replicon types were identified, with IncFIB and IncFII detected in 435 (75.4%) and 410 (71.1%) isolates, respectively (Fig. 2 and Table S6). Furthermore, the prevalence of replicons between isolates with ESBL and non-ESBL phenotypes was different for IncFIA (61.3% versus 38%, respectively; *P* = 0.041) and IncHI (10.8% versus 34%, respectively; *P* < 0.001).

10.1128/msphere.00471-22.7 TABLE S6 Plasmid replicon types identified from WGS analysis, stratified by ESBL phenotype. Statistical analyses were performed using Fisher’s exact test in R 3.0.1. Download Table S6, DOCX file, 0.01 MB. Copyright © 2022 Jackson et al. 2022 Jackson et al. https://creativecommons.org/licenses/by/4.0/ This content is distributed under the terms of the Creative Commons Attribution 4.0 International license.

### Correlation analysis of antimicrobial resistance genes and determination of PPV between genotypes and nonsusceptible phenotypes within ESCR UPEC isolates.

**FIG 3:**
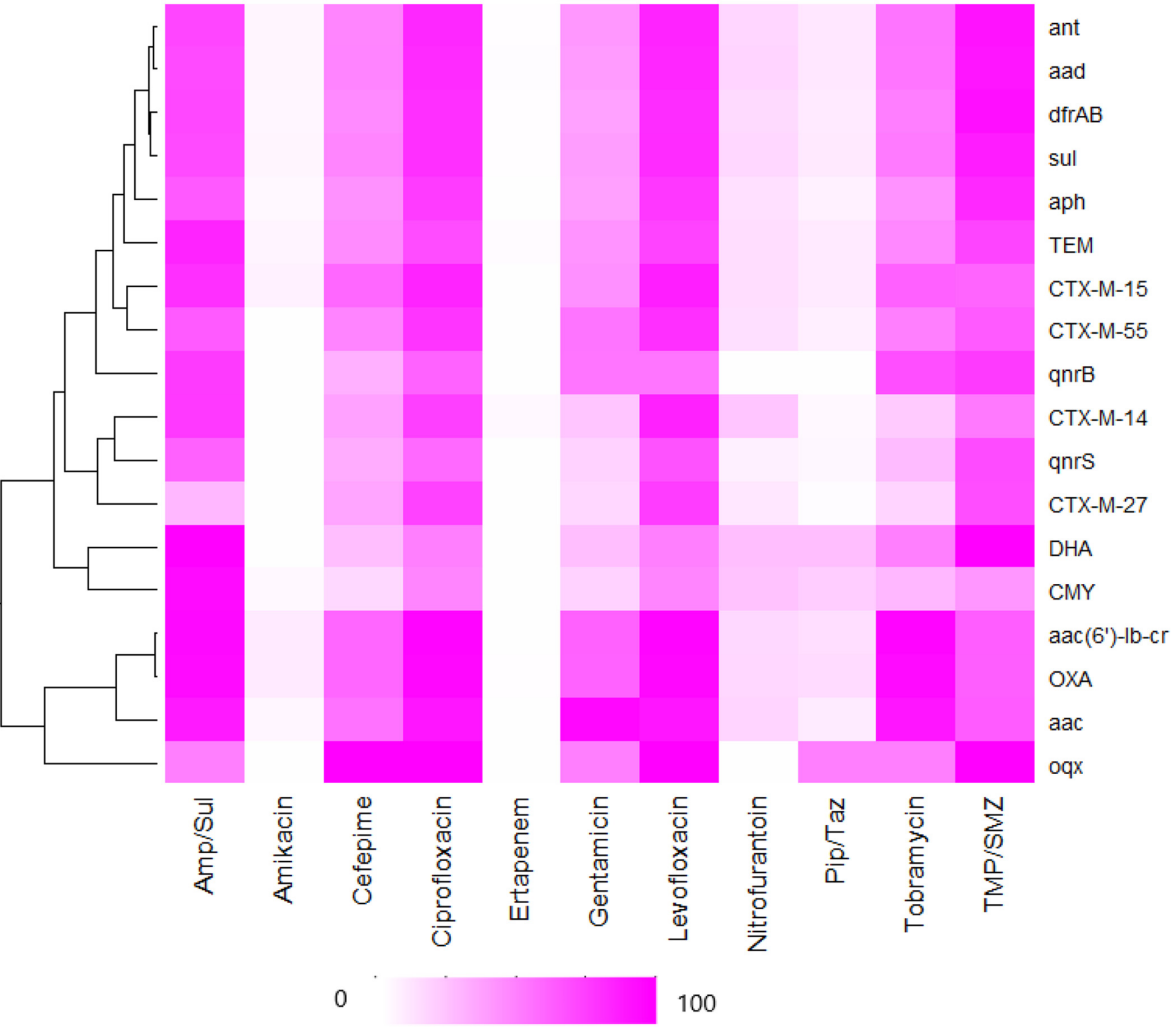
Heatmap displaying positive predictive value (PPV) of the presence of antibiotic resistance genes and phenotypic nonsusceptibility to antimicrobial agents used in the treatment of UTI. The PPV of each genotype-phenotype classification was calculated and visually displayed in the form of a heatmap. A PPV of 0 is shown in white, whereas a PPV of 1 is dark pink. Ninety-five percent confidence intervals were calculated by bootstrapping. Hierarchical clustering was applied to the genes included in this analysis. The heatmap was created with the heatmap package using R 3.0.1. Amp/Sul, ampicillin-sulbactam; Pip/Taz, piperacillin-tazobactam; TMP/SMZ, trimethoprim-sulfamethoxazole.

**FIG 4:**
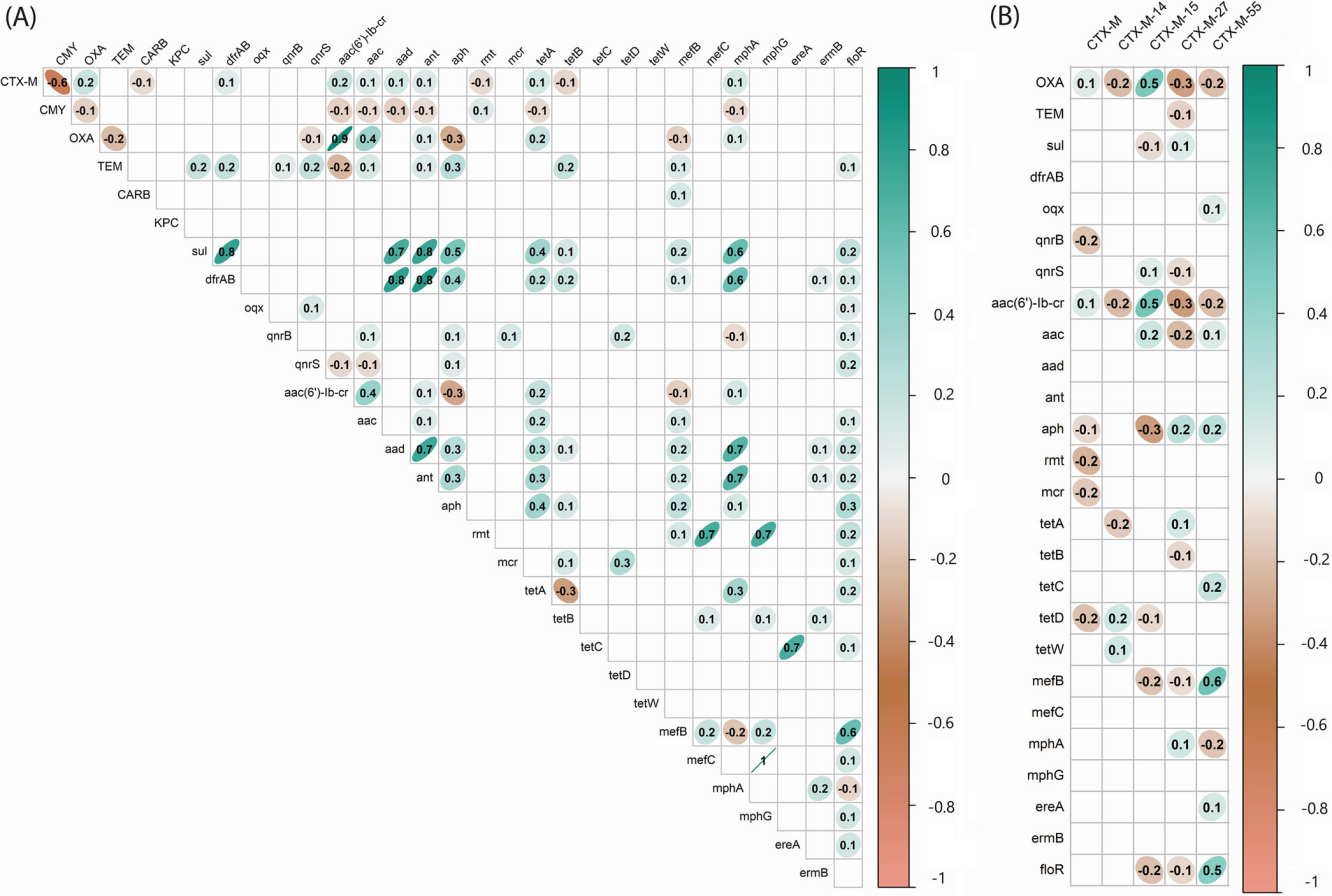
Correlation between β-lactamase genes and horizontally acquired resistance determinants, detected in ESCR UPEC from WGS analysis. (A) Resistance gene data were analyzed using the Phi correlation coefficient, for all ESCR UPEC isolates (*n* = 577). Genes encoding proteins with the same mechanism of action were grouped according to their class. (B) To examine relationships between common variants of *bla*_CTX-M_ and other horizontally acquired resistance genes, we analyzed the 527 isolates with ESBL phenotypes. Phi correlation coefficients can be interpreted as follows: 0, no relationship; ±<0.29, weak; between ±0.3 and ±0.49, moderate; between ±0.5 and ±0.99, strong; ±1, perfect. The ellipses surrounding correlation coefficients display the confidence regions of the distribution and strength of correlation. The figure was generated using the corrplot and ggplot2 functions in R 3.0.1.

We investigated the probability of a nonsusceptible phenotype given the presence of related and unrelated genotypes, by estimating the positive predictive value (PPV) (Fig. 3 and Table S7). The most common classes of horizontally acquired resistance genes identified from the WGS analysis were included in this analysis (prevalence of ≥5%) with the exceptions of *rmt* and *bla*_KPC_, which were present in <1% of the collection. The WGS data were analyzed alongside the susceptibility data for agents used to treat UTIs, including fluoroquinolones (ciprofloxacin and levofloxacin), aminoglycosides (tobramycin, gentamicin, and amikacin), β-lactams (ampicillin-sulbactam, piperacillin-tazobactam, cefepime, and ertapenem), nitrofurantoin, and trimethoprim-sulfamethoxazole. We also performed a gene-gene correlation analysis using the Phi correlation coefficient for all ESCR UPEC isolates (*n* = 577) and for those with an ESBL phenotype (*n* = 527) (Fig. 4).

10.1128/msphere.00471-22.8 TABLE S7 Matrix displaying positive predictive values (PPVs) for resistance genes of interest versus antimicrobial nonsusceptibility. Isolates which displayed intermediate or resistant susceptibility in relation to CLSI breakpoints were grouped and described as “nonsusceptible” for this analysis. Abbreviations: Amp-Sul, ampicillin-sulbactam; Pip-Taz, piperacillin-tazobactam; TMP-SMZ, trimethoprim-sulfamethoxazole. Download Table S7, DOCX file, 0.02 MB. Copyright © 2022 Jackson et al. 2022 Jackson et al. https://creativecommons.org/licenses/by/4.0/ This content is distributed under the terms of the Creative Commons Attribution 4.0 International license.

The probability of nonsusceptible phenotypes given the presence of a recognized horizontally acquired resistance gene ranged from 0 to 1. When examining nonsusceptibility to fluoroquinolones, the presence of *bla*_CTX-M_, *bla*_TEM_, *sul*, *dfrA/B*, *aad*, *ant*, and *aph* was associated with PPVs that ranged from 0.7 to 0.89, whereas the presence of *bla*_OXA_ or *aac(6*′*)-Ib-cr* was associated with a PPV of ≥0.9 (Fig. 3 and Table S7). The genes *bla*_OXA_ and *aac(6*′*)-Ib-cr* were found to be strongly correlated with one another (Φ = 0.9), whereas *bla*_CTX-M-15_ was moderately correlated (Φ = 0.5) with both *bla*_OXA_ and *aac(6*′*)-Ib-cr*. Hierarchical cluster analysis of PPV revealed a strong clustering of *bla*_OXA_ and *aac(6*′*)-Ib-cr*. The genes *dfrA/B*, *aad*, and *ant* demonstrated a PPV of nonsusceptibility to trimethoprim-sulfamethoxazole of ≥0.9. The genes *sul*, *bla*_TEM_, *qnrB*, *qnrS*, and *aph* displayed PPVs ranging from 0.7 to 0.89 for nonsusceptibility to trimethoprim-sulfamethoxazole. The results of the gene correlation analysis displayed a moderate to strong positive correlation (Φ = 0.4 to 0.8) between trimethoprim-sulfamethoxazole resistance determinants *sul* and *dfrA/B* and the aminoglycoside resistance determinants *aad*, *ant*, and *aph* and a weak positive correlation (Φ = 0.2) with *bla*_TEM_ genes. The genes *bla*_OXA_, *aac(6*′*)-Ib-cr*, and *aac* each had a PPV of ≥0.9 for tobramycin nonsusceptibility, and *aac* had a PPV of ≥0.9 for gentamicin nonsusceptibility. In addition to the strong correlation with *aac(6*′*)-Ib-cr* (Φ = 0.9), *bla*_OXA_ had a moderate correlation with other *aac* genes (Φ = 0.4). None of the genes included in this analysis were strong predictors for nitrofurantoin nonsusceptibility.

### Antibiotic resistance genes as predictors of antibiotic nonsusceptibility and MDR within ESCR UPEC isolates.

We assessed the presence of common β-lactamase genes and other resistance genes as predictors of resistance to agents used to treat UTI. From the correlation and PPV analyses, an association between *bla*_CTX-M-15_, *bla*_OXA-1_, and *aac(6*′*)-Ib-cr* genes was identified. Therefore, we first calculated the risk ratio (RR) of the cooccurrence of these genes and antibiotic nonsusceptibility (intermediate or resistant to a given agent). The cooccurrence of *bla*_CTX-M-15_/*bla*_OXA-1_/*aac(6*′*)-Ib-cr* (found in 32.1% of ESBL phenotype isolates) increased the risk of nonsusceptibility to all agents included in the analysis, with the exception of nitrofurantoin and trimethoprim-sulfamethoxazole. The presence of these genes led to an 8-fold increase in risk of nonsusceptibility to amikacin (RR = 8), an aminoglycoside used in the empirical treatment of cUTI and a potential treatment for clinically confirmed ESCR UPEC UTIs. The cooccurrence of these genes also resulted in a 1.79-fold increase in the risk of MDR (RR = 1.79) (Table 3).

**TABLE 3:** Risk of nonsusceptibility (intermediate or resistant phenotype) associated with the cooccurrence of *bla*_CTX-M-15_, *bla*_OXA-1_, and *aac(6*′*)-Ib-cr* within isolates with an ESBL phenotype^*a*^

Antimicrobial agent	Risk ratio	95% confidence interval	*P*
Ampicillin-sulbactam	1.71	1.56–1.88	**<0.001**
Amikacin	8.08	2.72–23.98	**<0.001**
Cefepime	1.34	1.14–1.56	**0.001**
Ciprofloxacin	1.33	1.25–1.42	**<0.001**
Levofloxacin	1.26	1.19–1.34	**<0.001**
Gentamicin	2.46	2.00–3.04	**<0.001**
Nitrofurantoin	1.33	0.85–2.10	0.212
Piperacillin-tazobactam	3.41	1.70–6.87	**0.001**
Tobramycin	3.76	3.16–4.48	**<0.001**
Trimethoprim-sulfamethoxazole	0.99	0.85–1.14	0.923
MDR	1.79	1.63–1.98	**<0.001**

a Risk ratios were calculated using unconditional maximum-likelihood estimation, and 95% confidence intervals were calculated using normal approximation. Bold face highlights statistically significant results.

**FIG 5:**
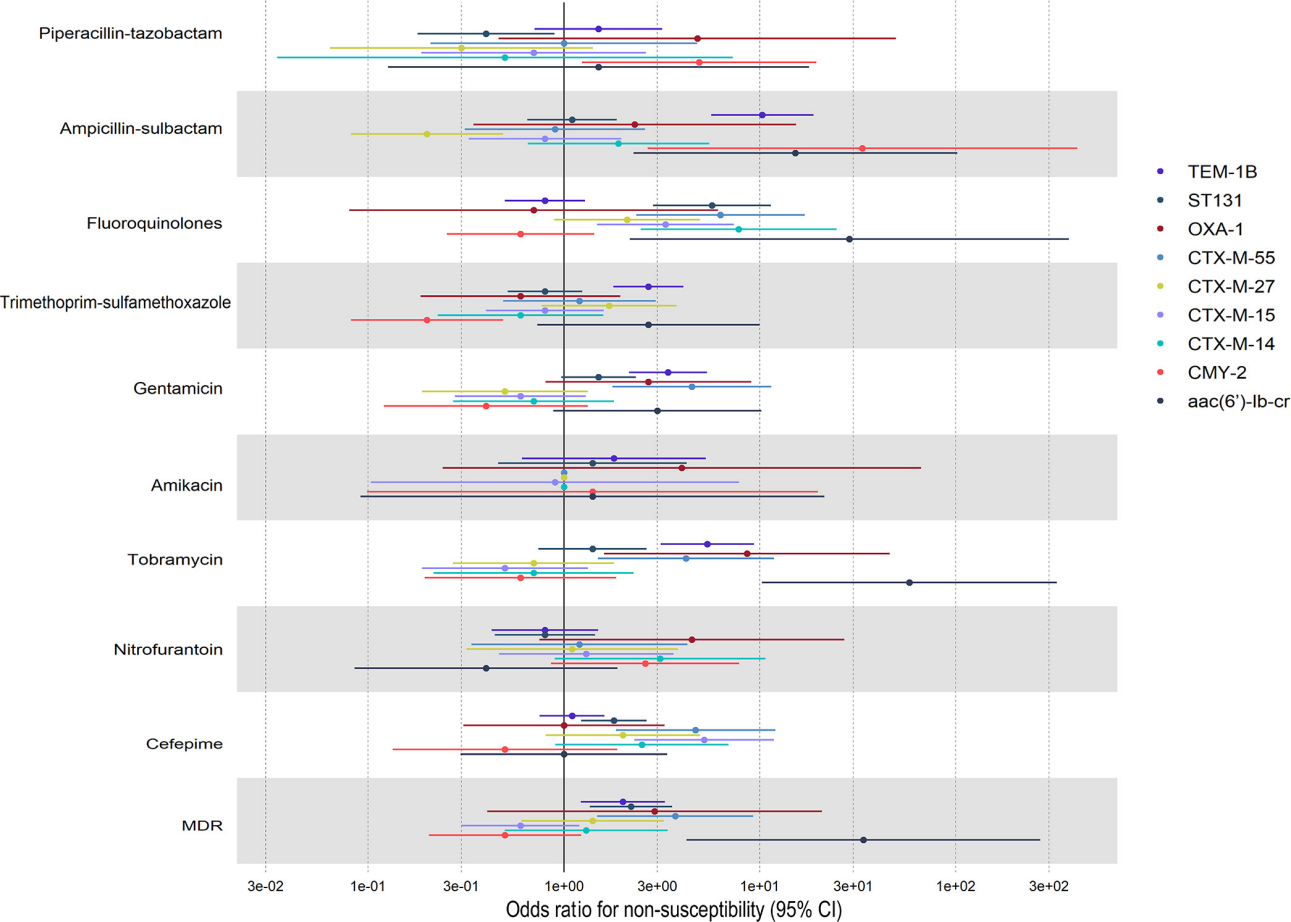
Forest plot displaying results of logistic regression analysis to assess the presence of resistance genes of interest and the ST131 lineage, as predictors of antibiotic nonsusceptibility and MDR in UTI isolates. Generalized linear model used a logit link function. Outcomes of the 10 models are binary (1 or 0), with 1 denoting nonsusceptibility or MDR status as defined as nonsusceptible to ≥3 classes of antimicrobial agents included in this analysis. Covariates in each model include ST131, *bla*_TEM-1B_, *bla*_OXA-1_, *bla*_CTX-M-55_, *bla*_CTX-M-27_, *bla*_CTX-M-15_, *bla*_CTX-M-14_, *bla*_CMY-2_, and *aac(6*′*)-lb-cr*.

To examine the individual risk of nonsusceptibility associated with the presence of individual genes while controlling for the effect of MLST, we conducted logistic regression. We defined the primary outcome of these models to be nonsusceptibility to antibiotics used in the treatment of UTI. We also included MDR as an outcome (Fig. 5 and Table S8). Our predictors of interest were the most prevalent *bla*_CTX-M_ variants identified, the most common pAmpC gene identified (*bla*_CMY-2_), and the narrow-spectrum β-lactamase genes *bla*_TEM-1B_ and *bla*_OXA-1_. We controlled for confounding by MLST by including ST131 classification in our model, as it was the predominant MDR-associated MLST (46% of isolates). We also included *aac(6*′*)-Ib-cr* in our model, due to the previously identified association of this gene with *bla*_OXA-1_ and *bla*_CTX-M-15_. Results indicate that the risk of antibiotic nonsusceptibility differed between the presence of *bla*_CTX-M_ variants and that of *bla*_CMY-2_ (piperacillin-tazobactam, trimethoprim-sulfamethoxazole, gentamicin, tobramycin, and cefepime), as well as between *bla*_CTX-M_ variants (ampicillin-sulbactam, fluoroquinolones, gentamicin, tobramycin, and cefepime). With the exception of *bla*_CTX-M-55_ (odds ratio [OR], 3.7; 95% confidence interval [CI], 1.5 to 9.4), the presence of *bla*_CTX-M_ or *bla*_CMY-2_ variants alone was not a predictor of MDR, and instead, *bla*_TEM-1B_ (OR, 2.0; 95% CI, 1.3 to 3.5), *aac(6*′*)-Ib-cr* (OR, 33.8; 95% CI, 4.9 to 313), or the ST131 genotype (OR, 2.2; 95% CI, 1.4 to 3.7) was identified as a predictor of MDR within ESCR UPEC isolates. The only predictor of nonsusceptibility to the carbapenem-sparing agent piperacillin-tazobactam was *bla*_CMY-2_ (OR, 4.9; 95% CI, 1.3 to 20.4). Detailed results of the regression analysis can be found in Table S8.

10.1128/msphere.00471-22.9 TABLE S8 Logistic regression analysis to assess the presence of common resistance genes as predictors of antibiotic nonsusceptibility and MDR in ESCR UPEC. Generalized linear model using a logit link function and the glm(family = binomial) function in R. Outcomes are binary (1 or 0), with 1 denoting nonsusceptibility or MDR status (defined as resistant to at least 1 agent in ≥3 classes of antimicrobial agents). The most common β-lactamase genes, the MLST type, ST-131, and the acetyltransferase gene *aac(6*′*)-Ib-cr* were included in the analysis. Abbreviations: Pip-Taz, piperacillin-tazobactam; FQ, fluoroquinolones; TMP-SMZ, trimethoprim-sulfamethoxazole; NIT, nitrofurantoin. Download Table S8, DOCX file, 0.02 MB. Copyright © 2022 Jackson et al. 2022 Jackson et al. https://creativecommons.org/licenses/by/4.0/ This content is distributed under the terms of the Creative Commons Attribution 4.0 International license.

An additional regression was performed, examining the association between the most prevalent gyrase and/or topoisomerase IV mutations (*gyrA*, D87N, S83L, and E84V; *parC*, E84V and S80I; *parE*, I529L, L416F, and S458A) and fluoroquinolone nonsusceptibility, controlling for the effects of resistance genes described previously, in addition to the lineage ST131 (Table S9). The results indicate that the ST131 lineage (OR, 3.06; 95% CI, 1.49 to 6.61; *P* = 0.003), *aac(6*′*)-Ib-cr* (OR, 56.48; 95% CI, 4.08 to 865.12; *P* = 0.003), and prevalent gyrase and/or topoisomerase IV mutations (OR, 16.42; 95% CI, 8.84 to 31.77; *P* < 0.001) were predictors for fluoroquinolone nonsusceptibility. No β-lactamase genes were identified as predictors for fluoroquinolone nonsusceptibility; however, *bla*_TEM-1B_ had a protective effect (OR, 0.36; 95% CI, 0.19 to 0.66; *P* = 0.001).

10.1128/msphere.00471-22.10 TABLE S9 Logistic regression analysis to assess the presence of common resistance genes as predictors of antibiotic nonsusceptibility to fluoroquinolones in ESCR UPEC. Generalized linear model used a logit link function and the glm(family = binomial) function in R. Outcomes are binary (1 or 0). Download Table S9, DOCX file, 0.01 MB. Copyright © 2022 Jackson et al. 2022 Jackson et al. https://creativecommons.org/licenses/by/4.0/ This content is distributed under the terms of the Creative Commons Attribution 4.0 International license.

### Pangenome and phylogenetic analysis.

**FIG 6:**
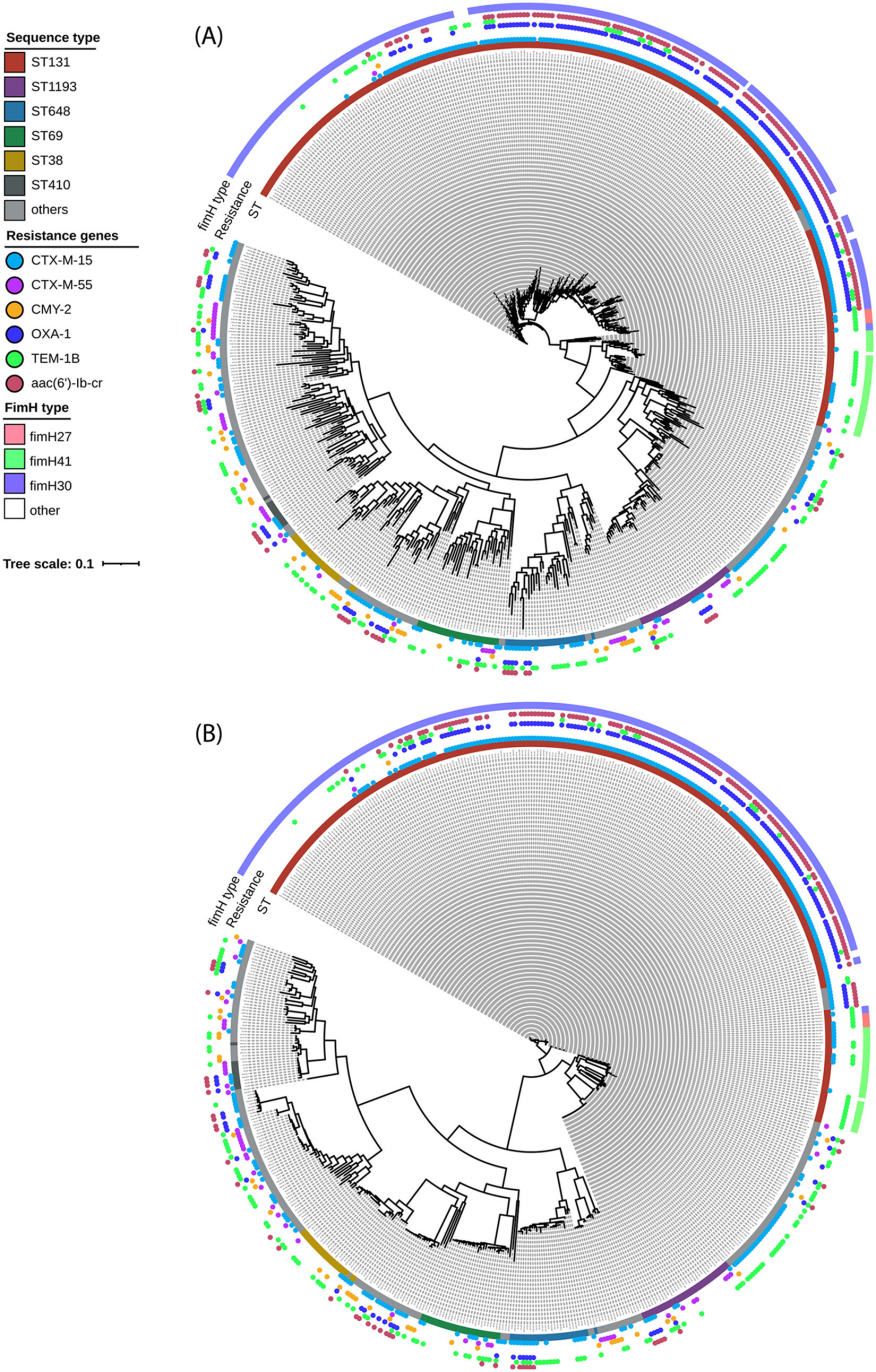
Maximum-likelihood phylogenetic trees of the ESCR UPEC isolates in this collection, displaying *fimH* type, MLST, and β-lactamase/resistance genes of interest. Trees display phylogenetic analysis based on the accessory genome (A) and SNP information (B) from pangenome analyses. Isolate sequence type is highlighted by the color-coded internal bar (“ST”), the presence of resistance genes of interest is denoted by the colored circles (“Resistance”), and the *fimH* type is displayed by the external colored bar framing the phylogenetic tree (“fimH type”).

WGS information for the ESCR UPEC isolates was subjected to pangenome and phylogenetic analysis (Fig. 6). A total of 32,586 genes constituted the pangenome. Of these, 2,967 (9%) were shared among more than 95% of the isolates (core genes) and 29,619 (91%) were distributed among subsets of the isolates (accessory genes). Of the latter, 26,437 genes were found in <15% of the isolates (cloud genes).

Maximum-likelihood phylogenies of the single nucleotide polymorphism (SNP) alignment of core genomes and the presence and absence of accessory genomes were obtained from FastTree (9) with the Jukes-Cantor model (Fig. 6). To assess the distribution of genes of interest, the most prevalent MLSTs were highlighted, alongside *fimH* type and the dominant β-lactamase genes conferring an ESCR phenotype (*bla*_CTX-M-15_, *bla*_CMY-2_). Genes previously identified as risk factors for MDR were also highlighted, including *bla*_CTX-M-55_ and *bla*_TEM-1B_ and the cooccurrence of *bla*_OXA-1_ and *aac(6*′*)-Ib-cr* with *bla*_CTX-M-15_.

Clustering based on MLST and clonal group was observed; for instance, in the ST131 clonal group, ST131, ST2279, ST8257, and ST8671 were present in a single cluster. Clustering based on *fimH* type was observed within ST131 phylogenies, with *fimH30* predominating. Regarding resistance genes, clustering based on the carriage of different classes of β-lactamase genes was observed in all MLSTs. Isolates with a cooccurrence of *bla*_CTX-M-15_/*bla*_OXA-1_/*aac(6*′*)-lb-cr* genes and isolates with only *bla*_CTX-M-15_ belong to different clades, a trend which is observed in all MLSTs. The results of this analysis also highlight that the majority of isolates with a cooccurrence of *bla*_CTX-M-15_/*bla*_OXA-1_/*aac(6*′*)-lb-cr* genes are associated with the ST131 lineage, as 76.5% of all isolates in which these three genes were detected were ST131. This cooccurrence was also observed in the emerging lineages ST1193 and ST648 and the MDR lineage ST410, as well as 12 other characterized MLSTs, and in isolates of unknown MLST. Isolates containing *bla*_CTX-M_-type genes belonged to different clades than those harboring *bla*_CMY_ variants. The *bla*_CTX-M-55_ gene was identified within 20 distinct MLSTs, with the emerging lineages ST1193 and ST774 harboring 14.6% and 16.7% of all *bla*_CTX-M-55_ genes detected, respectively. The *bla*_TEM-1B_ gene was also present in 35 (47.9%) of 73 characterized lineages identified in this collection.

## DISCUSSION

The primary aim of this study was to characterize the phenotypic antimicrobial susceptibility and underlying genotypes of ESCR UPEC isolates from patients with UTI in California, in addition to identifying genetic risk factors for nonsusceptibility and MDR. We observed a predominance of high-risk, MDR-associated ExPEC lineages ST131 (46%), ST69 (4.5%), and ST410 (1.7%), as well as emerging MDR lineages ST1193 and ST648 (collectively, 10% of isolates) (30–33). We also describe the frequency of β-lactamase genes, which underlie the ESCR phenotype. As expected, third-generation cephalosporin resistance was predominantly attributed to the presence of either ESBL (91.8%) or pAmpC β-lactamases (9%), and within these two groups the *bla*_CTX-M-15_ and *bla*_CMY-2_ genes were the most prevalent, respectively. This is unsurprising, as *bla*_CTX-M-15_ and *bla*_CMY-2_ genes predominate globally in ESCR-E. However, the emerging ESBL variants *bla*_CTX-M-27_ and *bla*_CTX-M-55_ were also identified in a notable proportion of isolates (16.8% and 8.3%, respectively) (34–38). The correlation analysis also highlighted an inverse correlation of the cooccurrence of ESBL and pAmpC-type β-lactamase genes, meaning that within this data set, the cooccurrence of these genes was unlikely; as a result, only 14 (2.3%) isolates contained both ESBL and pAmpC genes, and of these isolates, 13 (92.8%) retained an ESBL phenotype (as determined by ESBL confirmatory testing according to CLSI). This suggests that phenotypic ESBL testing is (for the most part) reliable. The potential cooccurrence of pAmpC and ESBL β-lactamases, which has been cited as a reason against the further use of this test, is rare at least within the study region of California (39).

Our results suggest that if, in clinical practice, the ESBL phenotype (or ESBL) could be identified by rapid diagnostics before susceptibility testing results are available, there is the potential to significantly reduce unnecessary UTI treatment escalation at empirical prescribing, particularly to the carbapenems. In addition, in our study population differences were seen between ESBL phenotype and non-ESBL phenotype ESCR UPEC isolates in their susceptibility to agents commonly used to treat UTIs. ESBL phenotype isolates were more frequently nonsusceptible to fluoroquinolones, and the prevalence of MDR among ESBL phenotype UPEC isolates was 1.7 times higher than that among non-ESBL phenotype UPEC isolates (*P* < 0.001). One limitation of this study is that because it was restricted to ESCR organisms, examining risk factors for MDR is subsequently not generalizable to all UPEC infections. However, these risk factors still provide the opportunity to reduce unnecessary treatment escalation for patients who screen positive for ESBL.

In the identification of genetic predictive factors for nonsusceptibility, the emerging ESBL variant *bla*_CTX-M-55_ and the lineage ST131 were identified as strong predictors of MDR within ESCR UPEC isolates. ST131 is recognized as a problematic MDR lineage globally, whereas MDR *Enterobacterales* harboring *bla*_CTX-M-55_ have been previously identified from clinical samples in China and France, in addition to poultry samples from Brazil (38, 40–44). The genotypic and phylogenetic analysis carried out in this study has revealed a strong correlation between *bla*_CTX-M-15_, *bla*_OXA-1_, and *aac(6*′*)-Ib-cr* among the ESCR UPEC isolates. These genes were commonly found together and were present in almost one-third (*n* = 169) of all ESBL phenotype isolates. This association has been identified previously in ESBL-producing E. coli isolates from the United Kingdom and Portugal and was shown to correlate with resistance to piperacillin-tazobactam, amoxicillin-clavulanic acid, and tobramycin (45, 46).

This research highlights the cooccurrence of *bla*_CTX-M-15_, *bla*_OXA-1_, and *aac(6*′*)-Ib-cr* within ESCR UPEC isolates as a risk factor for MDR, as well as for nonsusceptibility to agents used to treat UTI, including carbapenem-sparing agents (piperacillin-tazobactam, amikacin, and the fluoroquinolones). In fact, the cooccurrence of *bla*_CTX-M-15_/*bla*_OXA-1_/*aac(6*′*)-Ib-cr* was a strong risk factor for nonsusceptibility to amikacin. The gene *aac(6*′*)-Ib-cr* encodes a bifunctional aminoglycoside-modifying acetyltransferase, which has been shown to reduce susceptibility to tobramycin, kanamycin, and amikacin, in addition to the fluoroquinolones (47). This variant is characterized by the presence of two amino acid changes, at codons 102 (Trp→Arg) and 179 (Asp→Tyr), in comparison to its predecessor, *aac(6*′*)-Ib*, with these mutations conferring an extended spectrum of activity toward fluoroquinolones (48). Despite this, the results of the regression analysis showed that the presence of the *aac(6*′*)-Ib-cr* gene alone was not sufficient to confer clinical levels of resistance to amikacin, suggesting that other genetic mechanisms (efflux, 16S rRNA methylation, or interplay with other horizontally acquired aminoglycoside resistance mechanisms) (49) may contribute to amikacin resistance in isolates coharboring *bla*_CTX-M-15_/*bla*_OXA-1_/*aac(6*′*)-Ib-cr*; further investigation is needed to understand the mechanistic basis of amikacin nonsusceptibility in these isolates. However, *aac(6*′*)-Ib-cr* did increase the risk of nonsusceptibility to fluoroquinolone antibiotics, which was highlighted in the regression analysis in which we controlled for confounding by prevalent gyrase and/or topoisomerase IV mutations. Previous studies into the mechanistic basis of *aac(6*′*)-Ib-cr* have shown that this gene alone cannot confer clinical levels of resistance to fluoroquinolones. Therefore, there may be interplay between *aac(6*′*)-Ib-cr* and other fluoroquinolone resistance mechanisms within isolates from this collection which were not controlled for in this analysis (50). Further investigation is required to understand the contribution of *aac(6*′*)-Ib-cr* to fluoroquinolone nonsusceptibility within the isolates from this study. Lastly, the presence of *bla*_OXA-1_ in ESBL producers has been recognized previously to reduce susceptibility to penicillin-inhibitor combinations. It has been postulated that ESBL isolates co-expressing *bla*_OXA-1_ were responsible for the inferiority of piperacillin-tazobactam, compared to carbapenems, in the treatment of ESCR infections in the MERINO trial (51).

In conclusion, this study provides a regional description of prevalent MDR UPEC lineages, phenotypic coresistance profiles, and resistance determinants in ESCR UPEC isolates. This study highlights targets for improved antimicrobial resistance surveillance and helps identify putative genes underlying the ESCR phenotype within clinical isolates in California. Elucidating the specific β-lactamase genes present in these MDR organisms increases our understanding of associated coresistance profiles. Our results suggest the cooccurrence of *bla*_CTX-M-15_/*bla*_OXA-1_/*aac(6*′*)-Ib-cr* genes and the occurrence of *bla*_CTX-M-55_ constitute important risk factors for MDR in ESCR UPEC. Identification of these markers, in addition to ESBL phenotype, could inform empirical treatment decisions, including targeted carbapenem-sparing strategies, for ESCR UTIs. This would not only promote antibiotic stewardship but improve treatment outcomes. Antimicrobial resistance is now recognized as a leading cause of mortality worldwide (52); therefore, sustained efforts must be made to curb unnecessary antibiotic use through improved prescribing practices, to alleviate the public health burden of drug-resistant infections.

## MATERIALS AND METHODS

### Clinical samples and isolate collection.

Between February and October of 2019, we collected E. coli bacteria isolated from urine specimens (UPEC) from 6 different clinical laboratory sites across California (4 sites in northern and 2 sites in southern California). Only E. coli isolates growing at clinically significant thresholds as determined per standard operating procedures at each site and isolates which tested nonsusceptible (intermediate or resistant MIC value) to third-generation cephalosporins (cefotaxime, ceftazidime, or ceftriaxone; ESCR) according to CLSI interpretive criteria (53) were selected for further analysis (*n* = 577 isolates). Duplicate patient samples were removed from the analysis.

### Susceptibility testing and ESBL confirmatory testing.

Susceptibility testing was conducted by the respective clinical laboratories from which isolates were collected; MICs were determined in accordance with CLSI guidelines, and MIC determination was performed on the MicroScan WalkAway (Neg/Urine Combo type 73 panel; Beckman Coulter, Brea, CA, USA), Trek Sensititre (GN6F panel; Thermo Scientific, Emeryville, CA, USA), or Vitek 2 (AST-GN90 panel; bioMérieux, Inc., Durham, NC, USA) panel. MIC results were interpreted as susceptible, intermediate, or resistant according to interpretative criteria outlined in CLSI guidelines (53). Susceptibilities to the following antimicrobial agents were included in this study: cefotaxime, ceftazidime, ceftriaxone, cefepime, ampicillin-sulbactam, piperacillin-tazobactam, ertapenem, amikacin, tobramycin, gentamicin, ciprofloxacin, levofloxacin, nitrofurantoin, and trimethoprim-sulfamethoxazole. If susceptibility information was missing for a given isolate, susceptibility testing was carried out using the disk diffusion method, in accordance with CLSI guidelines (53). An MDR isolate was defined as one that tested resistant to at least 1 agent in ≥3 classes of antimicrobial agents included in this analysis: β-lactams, fluoroquinolones, aminoglycosides, trimethoprim-sulfamethoxazole, and nitrofurantoin.

ESBL confirmatory testing was performed with the disk diffusion method (using disks containing cefotaxime, cefotaxime plus clavulanic acid, ceftazidime, and ceftazidime plus clavulanic acid) per CLSI guidelines (53). A positive or negative ESBL confirmatory testing result was used to group isolates as ESBL phenotype or non-ESBL phenotype, respectively. For quality control, the CLSI-recommended reference strains ATCC 25922 and ATCC 700603 were tested alongside clinical isolates (53).

### Whole-genome sequencing and genome assembly.

DNA was extracted from E. coli isolates, and the libraries were prepared with the Nextera DNA Flex library prep kit (Illumina, USA), and then libraries were sequenced on the MiSeq platform (Illumina, USA). MiSeq reads were screened and trimmed based on length and quality with BBDuk (version 1.0) under the default setting (54). The trimming process also removed residual adapter sequences. Quality check of individual FASTQ files was conducted with FastQC (55). *De novo* assembly of trimmed paired reads for the libraries was performed with Unicycler (version 0.4.8) under the setting “--min_fasta_length 500” to remove contigs less than 500 bp (56). The number of reads used in each assembly was sufficient to give a minimum of 25-fold coverage, averaged across all contigs. A maximum of 45-fold coverage was obtained. Contigs that were less than 500 bp in length and with fewer than 80% high-quality base calls were eliminated from subsequent analysis.

Annotation was performed on all the assembled genomes with Prokka (version 1.14.0) (57). Full assemblies were uploaded to the Batch Processing portal at the Center for Genomic Epidemiology to confirm species and identify plasmid replicons, antibiotic resistance, and virulence genes (58). *fimH* types were identified with the FimTyper database (accessed April 2020) (59). All identifications were done with Abricate (version 1.0.1) (https://github.com/tseemann/abricate) with a 95% identity threshold across the reference sequences. Multilocus sequence typing was determined with mlst (version 2.19.0) (https://github.com/tseemann/mlst) based on the seven-gene Achtman scheme (60, 61).

In the description of the β-lactamase genes identified from our WGS analysis, we defined β-lactamases other than ESBL, pAmpC, and carbapenemases (such as non-ESBL TEM, SHV, and OXA variants) as having a “narrow spectrum” of activity.

### Bioinformatics and phylogenetic analysis.

A pangenome of all *de novo* assemblies (*n* = 577) and of ST131 (*n* = 267) was constructed with Roary (version 3.13.0) with a 95% identity cutoff (62). Here, genes present in ≥95% of the cohort isolates were defined as “core” and constituting the core portion of the metagenome, and genes present in less than 95% were defined as accessory and constituting the accessory portion of the metagenome. Accessory genes present in <15% of the cohort isolates were defined as cloud genes. A concatenated core coding sequence (CDS) alignment was made from the Roary output, and we extracted single nucleotide polymorphism (SNP) information with SNP-sites with the default option (63). Phylogenetic trees were constructed with FastTree (version 2.1.10) with the maximum-likelihood method with the Jukes-Cantor model based on the SNP alignment and the presence and absence of accessory genes from the Roary output (64). Visualization was done with iToL, version 6.1.2 (http://itol.embl.de).

### Statistical analysis and data visualization.

Data visualization, generation of graphs, and statistics were performed with R 3.0.1. Adjustment for multiple comparisons was done using the Bonferroni method (65) (https://www.r-project.org/). The R packages and functions used in this study included ggplot2 (https://www.rdocumentation.org/packages/ggplot2/versions/3.3.5), corrplot (https://www.rdocumentation.org/packages/corrplot/versions/0.2-0/topics/corrplot), heatmap (https://www.rdocumentation.org/packages/stats/versions/3.6.2/topics/heatmap), chordDiagram (https://www.rdocumentation.org/packages/circlize/versions/0.4.13/topics/chordDiagram), glm (https://www.rdocumentation.org/packages/SparkR/versions/2.1.2/topics/glm), and VennDiagram (https://cran.r-project.org/web/packages/VennDiagram/VennDiagram.pdf). Figures were resized or relabeled on Adobe Illustrator (version 25.0.01) (Adobe Systems Incorporated, San Jose, CA, USA).

### Data availability.

The Illumina sequences generated in this study are deposited and are available from the National Center for Biotechnology Information’s database, under the BioProject ID: PRJNA891712.
