# Supplementary material for: Genetic Predictive Factors for Nonsusceptible Phenotypes and Multidrug Resistance in Expanded-Spectrum Cephalosporin-Resistant Uropathogenic Escherichia coli from a Multicenter Cohort: Insights into the Phenotypic and Genetic Basis of Coresistance
Source: mSphere. 2022 Nov 15;7(6):e00471-22. doi: 10.1128/msphere.00471-22 (PMC9769571; doi:10.1128/msphere.00471-22)
Supplement: FIG S1 [file msphere.00471-22-s0010.docx]

***Supplementary information***

**
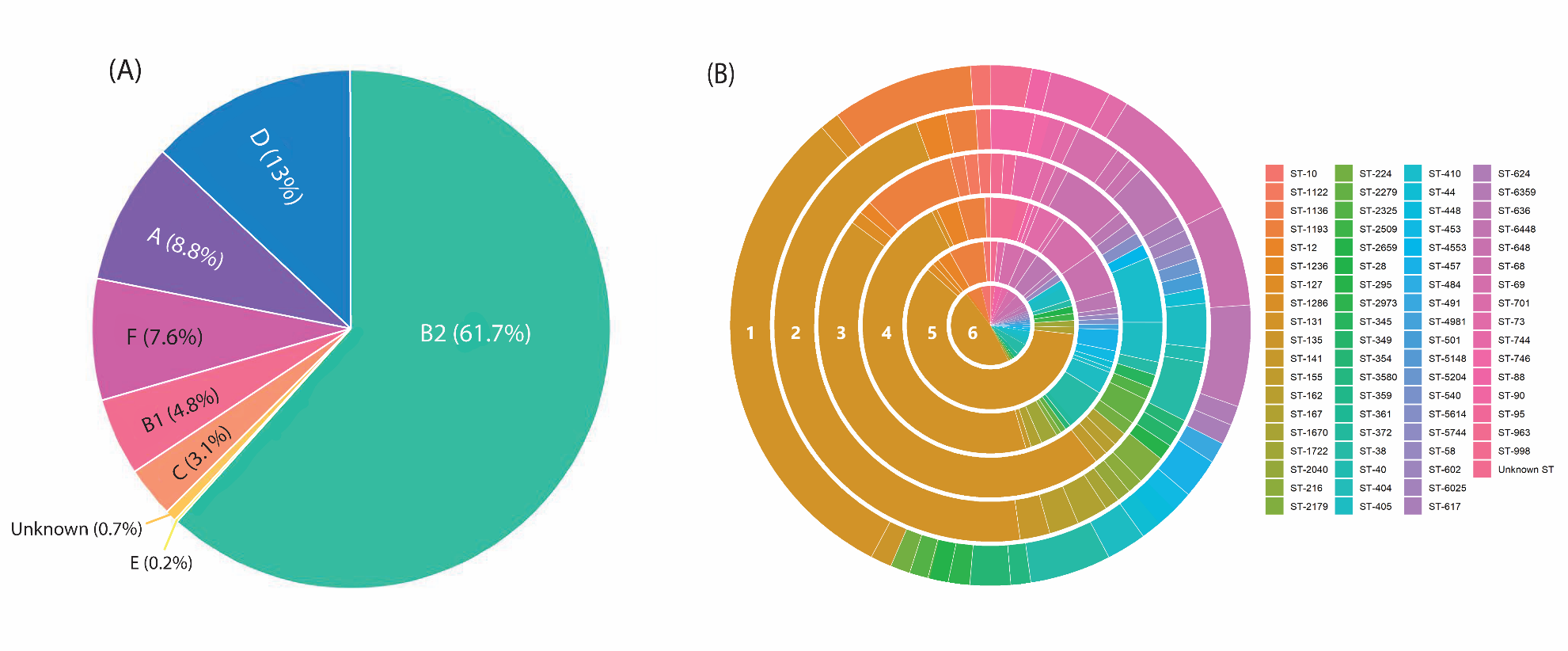
**

**Supplementary Figure S1:** (A) Proportions of phylogroups identified, and (B) pie plot displaying prevalence of MLSTs, sorted by clinical laboratory site (Sites 1-6). Diagrams were created using ggplot2 in R 3.0.1.
