# Supplementary material for: Genetic Predictive Factors for Nonsusceptible Phenotypes and Multidrug Resistance in Expanded-Spectrum Cephalosporin-Resistant Uropathogenic Escherichia coli from a Multicenter Cohort: Insights into the Phenotypic and Genetic Basis of Coresistance
Source: mSphere. 2022 Nov 15;7(6):e00471-22. doi: 10.1128/msphere.00471-22 (PMC9769571; doi:10.1128/msphere.00471-22)
Supplement: TABLE S1 [file msphere.00471-22-s0001.docx]

**Supplementary Table S1:** Antimicrobial non-susceptibility (susceptibility categorized as intermediate or resistant in relation to CLSI breakpoints), stratified by phenotypic ESBL status. Statistical analyses were performed using Fisher’s exact test in R 3.0.1. In the table, the ‘-’ symbol denotes that the sample size was not sufficient to generate a *p* value, whereas ‘NS’ indicates a non-significant result.

|  |  |  |  |  |
| --- | --- | --- | --- | --- |
| **Antimicrobial non-susceptibility** | **Non-ESBL (N=50)** | **ESBL (N=527)** | **Overall (N=577)** | ***p*** |
| Trimethoprim/sulfamethoxazole | 22 (44.0%) | 328 (62.2%) | 350 (60.7%) | NS |
| Nitrofurantoin | 10 (20.0%) | 68 (12.9%) | 78 (13.5%) | NS |
| Levofloxacin | 20 (40.0%) | 444 (84.3%) | 464 (80.4%) | **<0.001** |
| Ciprofloxacin | 20 (40.0%) | 431 (81.8%) | 451 (78.2%) | **<0.001** |
| Tobramycin | 11 (22.0%) | 258 (49.0%) | 269 (46.6%) | **0.004** |
| Gentamicin | 11 (22.0%) | 197 (37.4%) | 208 (36.0%) | NS |
| Amikacin | 0 (0%) | 19 (3.6%) | 19 (3.3%) | NS |
| Ertapenem | 1 (2.0%) | 2 (0.4%) | 3 (0.5%) | - |
| Piperacillin/tazobactam | 9 (18.0%) | 31 (5.9%) | 40 (6.9%) | NS |
| Ampicillin/sulbactam | 44 (88.0%) | 366 (69.4%) | 410 (71.1%) | NS |
| Cefepime | 4 (8.0%) | 269 (51.0%) | 273 (47.3%) | **<0.001** |
| Cefotaxime | 39 (78.0%) | 525 (99.6%) | 564 (97.7%) | **<0.001** |
| Ceftriaxone | 49 (98.0%) | 525 (99.6%) | 574 (99.5%) | NS |
| Ceftazidime | 42 (84.0%) | 377 (71.5%) | 419 (72.6%) | NS |
| MDR | 20 (40.0%) | 368 (69.8%) | 388 (67.2%) | **<0.001** |
