# Supplementary material for: Genetic Predictive Factors for Nonsusceptible Phenotypes and Multidrug Resistance in Expanded-Spectrum Cephalosporin-Resistant Uropathogenic Escherichia coli from a Multicenter Cohort: Insights into the Phenotypic and Genetic Basis of Coresistance
Source: mSphere. 2022 Nov 15;7(6):e00471-22. doi: 10.1128/msphere.00471-22 (PMC9769571; doi:10.1128/msphere.00471-22)
Supplement: TABLE S2 [file msphere.00471-22-s0002.docx]

|  | **MLST** | | | | | | |
| --- | --- | --- | --- | --- | --- | --- | --- |
| **Antimicrobial non-susceptibility** | ST131 (N=267) | ST1193 (N=32) | ST38 (N=22) | ST636 (N=18) | ST648 (N=26) | ST69 (N=26) |  |
| Trimethoprim/sulfamethoxazole | 161 (60.3%) | 22 (68.8%) | 12 (54.5%) | 14 (77.8%) | 16 (61.5%) | 17 (65.4%) |  |
| Nitrofurantoin | 32 (12.0%) | 3 (9.4%) | 4 (18.2%) | 0 (0%) | 10 (38.5%) | 0 (0%) |  |
| Levofloxacin | 252 (94.4%) | 32 (100%) | 11 (50.0%) | 8 (44.4%) | 26 (100%) | 12 (46.2%) |  |
| Ciprofloxacin | 253 (94.8%) | 32 (100%) | 10 (45.5%) | 5 (27.8%) | 26 (100%) | 10 (38.5%) |  |
| Tobramycin | 160 (59.9%) | 10 (31.3%) | 0 (0%) | 2 (11.1%) | 14 (53.8%) | 7 (26.9%) |  |
| Gentamicin | 116 (43.4%) | 9 (28.1%) | 2 (9.1%) | 0 (0%) | 9 (34.6%) | 6 (23.1%) |  |
| Amikacin | 13 (4.9%) | 1 (3.1%) | 0 (0%) | 0 (0%) | 1 (3.8%) | 0 (0%) |  |
| Ertapenem | 2 (0.7%) | 0 (0%) | 0 (0%) | 0 (0%) | 1 (3.8%) | 0 (0%) |  |
| Piperacillin/tazobactam | 17 (6.4%) | 0 (0%) | 0 (0%) | 0 (0%) | 3 (11.5%) | 1 (3.8%) |  |
| Ampicillin/sulbactam | 195 (73.0%) | 18 (56.3%) | 12 (54.5%) | 11 (61.1%) | 23 (88.5%) | 13 (50.0%) |  |
| Cefepime | 13 (40.6%) | 154 (57.7%) | 9 (40.9%) | 6 (33.3%) | 14 (53.8%) | 7 (26.9%) |  |
| Cefotaxime | 260 (97.4%) | 31 (96.9%) | 22 (100%) | 18 (100%) | 26 (100%) | 24 (92.3%) |  |
| Ceftriaxone | 266 (99.6%) | 32 (100%) | 22 (100%) | 18 (100%) | 26 (100%) | 26 (100%) |  |
| Ceftazidime | 184 (68.9%) | 25 (78.1%) | 15 (68.2%) | 13 (72.2%) | 17 (65.4%) | 19 (73.1%) |  |
| MDR | 218 (81.6%) | 25 (78.1%) | 8 (36.4%) | 0 (0%) | 19 (73.1%) | 9 (34.6%) |  |
| ESBL phenotype | 260 (97.4%) | 29 (90.6%) | 20 (90.9%) | 18 (100%) | 25 (96.2%) | 22 (84.6%) |  |

**Supplementary Table S2:** Antimicrobial non-susceptibility (susceptibility categorized as intermediate or resistant in relation to CLSI breakpoints), stratified by the 6 most common MLSTs identified in this collection.
