# Supplementary material for: Genetic Predictive Factors for Nonsusceptible Phenotypes and Multidrug Resistance in Expanded-Spectrum Cephalosporin-Resistant Uropathogenic Escherichia coli from a Multicenter Cohort: Insights into the Phenotypic and Genetic Basis of Coresistance
Source: mSphere. 2022 Nov 15;7(6):e00471-22. doi: 10.1128/msphere.00471-22 (PMC9769571; doi:10.1128/msphere.00471-22)
Supplement: TABLE S3 [file msphere.00471-22-s0003.docx]

**Supplementary Table S3:** Antimicrobial resistance profiles, stratified by phenotypic ESBL status. Statistical analyses were performed using Fisher’s exact test in R 3.0.1. Abbreviations are defined as the following, BL = β-lactam, N = nitrofurantoin, FQ = fluoroquinolones, AG = aminoglycosides, TS = trimethoprim/sulfamethoxazole. In the table, the ‘-’ symbol denotes that the sample size was not sufficient to generate a *p* value, whereas ‘NS’ indicates a non-significant result.

| **Resistance classes** | **Non-ESBL (N=50)** | **ESBL (N=527)** | **Overall (N=577)** | ***p*** |
| --- | --- | --- | --- | --- |
| BL | 18 (36%) | 43 (8.1%) | 61 (10.6%) | **<0.001** |
| BL, N | 3 (6.0%) | 3 (0.6%) | 6 (1.0%) | NS |
| BL, FQ | 2 (4%) | 70 (13.3%) | 72 (12.5%) | NS |
| BL, TS | 6 (12%) | 42 (8%) | 48 (8.3%) | NS |
| BL, AG | 1 (2%) | 1 (0.2%) | 2 (0.3%) | NS |
| BL, FQ, N | 0 (0%) | 2 (0.4%) | 2 (0.3%) | - |
| BL, FQ, AG | 4 (8%) | 76 (14.4%) | 80 (13.9%) | NS |
| BL, FQ, TS | 5 (10%) | 102 (19.4%) | 107 (18.6%) | NS |
| BL, AG, TS | 1 (2%) | 21 (4%) | 22 (3.9%) | NS |
| BL, AG, N, TS | 1 (2%) | 0 (0%) | 1 (0.2%) | - |
| BL, FQ, N, TS | 2 (4%) | 8 (1.5%) | 10 (1.7%) | NS |
| BL, FQ, AG, TS | 6 (12%) | 144 (27.3%) | 150 (26%) | NS |
| BL, FQ, AG, N | 0 (0%) | 4 (0.8%) | 4 (0.7%) | - |
| BL, FQ, AG, N, TS | 1 (2%) | 11 (2.1%) | 12 (2%) | NS |
