# Supplementary material for: Genetic Predictive Factors for Nonsusceptible Phenotypes and Multidrug Resistance in Expanded-Spectrum Cephalosporin-Resistant Uropathogenic Escherichia coli from a Multicenter Cohort: Insights into the Phenotypic and Genetic Basis of Coresistance
Source: mSphere. 2022 Nov 15;7(6):e00471-22. doi: 10.1128/msphere.00471-22 (PMC9769571; doi:10.1128/msphere.00471-22)
Supplement: TABLE S4 [file msphere.00471-22-s0004.docx]

**Supplementary Table S4:** Isolates containing β-lactamase genes identified from the WGS analysis, stratified by ESBL phenotype. Statistical analyses were performed using Fisher’s exact test in R 3.0.1. In the table, the ‘-’ symbol denotes that the sample size was not sufficient to generate a *p* value, whereas ‘NS’ indicates a non-significant result.

| **β-lactamase classification** | **Family** | **Activity** | **Gene** | **non-ESBL (N=50)** | **ESBL (N=527)** | **Total (N=577)** | ***p*** |
| --- | --- | --- | --- | --- | --- | --- | --- |
| **Class A** | **CTX-M** | ESBL | **Any CTX-M** | 6 (12.0%) | 521 (98.9%) | 527 (91.3%) | **<0.001** |
|  |  | ESBL | CTX-M-1 | 0 (0%) | 4 (0.8%) | 4 (0.7%) | - |
|  |  | ESBL | CTX-M-3 | 0 (0%) | 5 (0.9%) | 5 (0.9%) | NS |
|  |  | ESBL | CTX-M-14 | 0 (0%) | 44 (8.3%) | 44 (7.6%) | NS |
|  |  | ESBL | CTX-M-15 | 3 (6.0%) | 321 (60.9%) | 324 (56.2%) | **<0.001** |
|  |  | ESBL | CTX-M-24 | 0 (0%) | 1 (0.2%) | 1 (0.2%) | - |
|  |  | ESBL | CTX-M-27 | 2 (4.0%) | 95 (18%) | 97 (16.8%) | NS |
|  |  | ESBL | CTX-M-32 | 0 (0%) | 1 (0.2%) | 1 (0.2%) | - |
|  |  | ESBL | CTX-M-55 | 0 (0%) | 48 (9.1%) | 48 (8.3%) | NS |
|  |  | ESBL | CTX-M-64 | 0 (0%) | 1 (0.2%) | 1 (0.2%) | - |
|  |  | ESBL | CTX-M-65 | 0 (0%) | 6 (1.1%) | 6 (1.0%) | NS |
|  |  | ESBL | CTX-M-201 | 1 (2.0%) | 0 (0%) | 1 (0.2%) | - |
|  | **TEM** | ESBL | TEM-10 | 0 (0%) | 1 (0.2%) | 1 (0.2%) | - |
|  |  | ESBL | TEM-15 | 1 (2.0%) | 0 (0%) | 1 (0.2%) | - |
|  |  | Narrow-spectrum | **Any narrow-spectrum TEM** | 20 (40%) | 187 (35.48%) | 207 (44.9%) | NS |
|  |  | Narrow-spectrum | TEM-1A | 0 (0%) | 1 (0.2%) | 1 (0.2%) | - |
|  |  | Narrow-spectrum | TEM-1B | 17 (34.0%) | 152 (28.8%) | 169 (29.3%) | NS |
|  |  | Narrow-spectrum | TEM-1C | 1 (2.0%) | 3 (0.6%) | 4 (0.7%) | - |
|  |  | Narrow-spectrum | TEM-35 | 0 (0%) | 1 (0.2%) | 1 (0.2%) | - |
|  |  | Narrow-spectrum | TEM-76 | 0 (0%) | 1 (0.2%) | 1 (0.2%) | - |
|  |  | Narrow-spectrum | TEM-104 | 0 (0%) | 2 (0.4%) | 2 (0.3%) | - |
|  |  | Narrow-spectrum | TEM-176 | 1 (2.0%) | 0 (0%) | 1 (0.2%) | - |
|  |  | Narrow-spectrum | TEM-209 | 0 (0%) | 1 (0.2%) | 1 (0.2%) | - |
|  |  | Narrow-spectrum | TEM-214 | 0 (0%) | 1 (0.2%) | 1 (0.2%) | - |
|  |  | Narrow-spectrum | TEM-216 | 0 (0%) | 4 (0.8%) | 4 (0.7%) | - |
|  |  | Narrow-spectrum | TEM-220 | 1 (2.0%) | 0 (0%) | 1 (0.2%) | - |
|  |  | Narrow-spectrum | TEM-232 | 0 (0%) | 15 (2.8%) | 15 (2.6%) | NS |
|  |  | Narrow-spectrum | TEM-234 | 0 (0%) | 6 (1.1%) | 6 (1.0%) | NS |
|  | **SHV** | ESBL | SHV-12 | 0 (0%) | 1 (0.2%) | 1 (0.2%) | - |
|  |  | Narrow-spectrum | SHV-198 | 1 (2.0%) | 1 (0.2%) | 2 (0.3%) | - |
|  | **CARB** | Carbenicillinase | CARB-2 | 1 (2.0%) | 1 (0.2%) | 2 (0.3%) | - |
|  | **KPC** | Carbapenemase | KPC-2 | 0 (0%) | 1 (0.2%) | 1 (0.2%) | - |
| **Class C** | **CMY** | pAmpC cephalosporinase | **Any CMY** | 33 (66%) | 15 (2.85%) | 48 (8.31%) | **<0.001** |
|  |  | pAmpC cephalosporinase | CMY-2 | 30 (60.0%) | 10 (1.9%) | 40 (6.9%) | **<0.001** |
|  |  | pAmpC cephalosporinase | CMY-4 | 1 (2.0%) | 0 (0%) | 1 (0.2%) | - |
|  |  | pAmpC cephalosporinase | CMY-42 | 1 (2.0%) | 0 (0%) | 1 (0.2%) | - |
|  |  | pAmpC cephalosporinase | CMY-130 | 1 (2.0%) | 5 (0.9%) | 6 (1.0%) | NS |
|  | **DHA** | pAmpC cephalosporinase | DHA-1 | 1 (2.0%) | 2 (0.4%) | 3 (0.5%) | - |
|  |  | pAmpC cephalosporinase | DHA-6 | 1 (2.0%) | 0 (0%) | 1 (0.2%) | - |
| **Class D** | **OXA** | Narrow-spectrum | **Any OXA** | 4 (8.0%) | 181 (34.35%) | 185 (32.1%) | **0.004** |
|  |  | Narrow-spectrum | OXA-1 | 4 (8.0%) | 177 (33.6%) | 181 (31.4%) | **0.007** |
|  |  | Narrow-spectrum | OXA-9 | 0 (0%) | 1 (0.2%) | 1 (0.2%) | - |
|  |  | Narrow-spectrum | OXA-10 | 0 (0%) | 2 (0.4%) | 2 (0.3%) | - |
|  |  | Narrow-spectrum | OXA-320 | 0 (0%) | 1 (0.2%) | 1 (0.2%) | - |
