# Supplementary material for: Genetic Predictive Factors for Nonsusceptible Phenotypes and Multidrug Resistance in Expanded-Spectrum Cephalosporin-Resistant Uropathogenic Escherichia coli from a Multicenter Cohort: Insights into the Phenotypic and Genetic Basis of Coresistance
Source: mSphere. 2022 Nov 15;7(6):e00471-22. doi: 10.1128/msphere.00471-22 (PMC9769571; doi:10.1128/msphere.00471-22)
Supplement: TABLE S5 [file msphere.00471-22-s0005.docx]

**Supplementary Table S5:** Horizontally acquired resistance genes and mutations conferring antibiotic resistance, identified from WGS analysis, and stratified by ESBL phenotype. Statistical analyses were performed using Fisher’s exact test in R 3.0.1. In the table, the ‘-’ symbol denotes that the sample size was not sufficient to generate a *p* value, whereas ‘NS’ indicates a non-significant result.

| **Resistance conferred** (*gene groupings for correlation analysis*) | **Gene** | **non-ESBL (N=50)** | **ESBL (N=527)** | **Total (N=577)** | ***p*** |
| --- | --- | --- | --- | --- | --- |
| **Sulfonamides *(sul)*** | *sul1* | 16 (32.0%) | 270 (51.2%) | 286 (49.6%) | NS |
|  | *sul2* | 20 (40.0%) | 216 (41.0%) | 236 (40.9%) | NS |
|  | *sul3* | 2 (4.0%) | 30 (5.7%) | 32 (5.5%) | NS |
| **Trimethoprim *(dfrA/B)*** | *dfrA1* | 3 (6.0%) | 27 (5.1%) | 30 (5.2%) | NS |
|  | *dfrA5* | 1 (2.0%) | 9 (1.7%) | 10 (1.7%) | NS |
|  | *dfrA8* | 1 (2.0%) | 2 (0.4%) | 3 (0.5%) | - |
|  | *dfrA12* | 7 (14.0%) | 33 (6.3%) | 40 (6.9%) | NS |
|  | *dfrA14* | 3 (6.0%) | 39 (7.4%) | 42 (7.3%) | NS |
|  | *dfrA16* | 0 (0%) | 1 (0.2%) | 1 (0.2%) | - |
|  | *dfrA17* | 11 (22.0%) | 236 (44.8%) | 247 (42.8%) | NS |
|  | *dfrA19* | 0 (0%) | 2 (0.4%) | 2 (0.3%) | - |
|  | *dfrA27* | 0 (0%) | 3 (0.6%) | 3 (0.5%) | - |
|  | *dfrA32* | 0 (0%) | 1 (0.2%) | 1 (0.2%) | - |
|  | *dfrB4* | 0 (0%) | 1 (0.2%) | 1 (0.2%) | - |
| **MDR efflux pump *(oqx)*** | *oqxAB* | 0 (0%) | 2 (0.4%) | 2 (0.3%) | - |
| **Fluoroquinolones *(qnrB, qnrS)*** | *qnrB1* | 0 (0%) | 4 (0.8%) | 4 (0.7%) | - |
|  | *qnrB2* | 0 (0%) | 1 (0.2%) | 1 (0.2%) | - |
|  | *qnrB4* | 1 (2.0%) | 2 (0.4%) | 3 (0.5%) | - |
|  | *qnrB19* | 0 (0%) | 5 (0.9%) | 5 (0.9%) | NS |
|  | *qnrS1* | 2 (4.0%) | 29 (5.5%) | 31 (5.4%) | NS |
|  | *qnrS2* | 0 (0%) | 3 (0.6%) | 3 (0.5%) | - |
|  | *mdfA* | 50 (100%) | 527 (100%) | 577 (100%) | - |
|  | *gyrA* mutation D87G | 0 (0%) | 2 (0.4%) | 2 (0.3%) | - |
|  | *gyrA* mutation D87N | 16 (32.0%) | 361 (68.5%) | 377 (65.3%) | **<0.001** |
|  | *gyrA* mutation D87Y | 1 (2.0%) | 9 (1.7%) | 10 (1.7%) | NS |
|  | *gyrA* mutation S83A | 0 (0%) | 1 (0.2%) | 1 (0.2%) | - |
|  | *gyrA* mutation S83L | 18 (36.0%) | 438 (83.1%) | 456 (79.0%) | **<0.001** |
|  | *parC* mutation A56T | 0 (0%) | 9 (1.7%) | 9 (1.6%) | NS |
|  | *parC* mutation E84G | 1 (2.0%) | 11 (2.1%) | 12 (2.1%) | NS |
|  | *parC* mutation E84V | 5 (10.0%) | 229 (43.5%) | 234 (40.6%) | **<0.001** |
|  | *parC* mutation S57T | 0 (0%) | 3 (0.6%) | 3 (0.5%) | - |
|  | *parC* mutation S80I | 17 (34.0%) | 382 (72.5%) | 399 (69.2%) | **<0.001** |
|  | *parC* mutation S80R | 0 (0%) | 4 (0.8%) | 4 (0.7%) | - |
|  | *parE* mutation E460D | 0 (0%) | 2 (0.4%) | 2 (0.3%) | - |
|  | *parE* mutation I355T | 2 (4.0%) | 3 (0.6%) | 5 (0.9%) | NS |
|  | *parE* mutation I529L | 6 (12.0%) | 247 (46.9%) | 253 (43.8%) | **<0.001** |
|  | *parE* mutation L416F | 5 (10.0%) | 33 (6.3%) | 38 (6.6%) | NS |
|  | *parE* mutation L445H | 1 (2.0%) | 1 (0.2%) | 2 (0.3%) | - |
|  | *parE* mutation S458A | 5 (10.0%) | 72 (13.7%) | 77 (13.3%) | NS |
|  | *parE* mutation S458T | 0 (0%) | 4 (0.8%) | 4 (0.7%) | - |
| **Fluoroquinolone/aminoglycoside** | *aac(6')-Ib-cr* | 4 (8.0%) | 182 (34.5%) | 186 (32.2%) | **0.004** |
| **Aminoglycosides** ***(aac, aad, ant, aph, rmt)*** | *aac(3)-IIa* | 2 (4.0%) | 137 (26.0%) | 139 (24.1%) | **0.026** |
|  | *aac(3)-IId* | 7 (14.0%) | 55 (10.4%) | 62 (10.7%) | NS |
|  | *aac(3)-IIe* | 1 (2.0%) | 2 (0.4%) | 3 (0.5%) | - |
|  | *aac(3)-IVa* | 0 (0%) | 2 (0.4%) | 2 (0.3%) | - |
|  | *aac(3)-VIa* | 0 (0%) | 1 (0.2%) | 1 (0.2%) | - |
|  | *aadA1* | 1 (2.0%) | 28 (5.3%) | 29 (5.0%) | NS |
|  | *aadA2* | 8 (16.0%) | 39 (7.4%) | 47 (8.1%) | NS |
|  | *aadA3* | 2 (4.0%) | 20 (3.8%) | 22 (3.8%) | NS |
|  | *aadA5* | 8 (16.0%) | 229 (43.5%) | 237 (41.1%) | NS |
|  | *aadA8b* | 2 (4.0%) | 10 (1.9%) | 12 (2.1%) | NS |
|  | *aadA11* | 0 (0%) | 1 (0.2%) | 1 (0.2%) | - |
|  | *aadA12* | 1 (2.0%) | 1 (0.2%) | 2 (0.3%) | NS |
|  | *aadA15* | 1 (2.0%) | 22 (4.2%) | 23 (4.0%) | NS |
|  | *aadA16* | 0 (0%) | 3 (0.6%) | 3 (0.5%) | - |
|  | *aadA17* | 0 (0%) | 3 (0.6%) | 3 (0.5%) | - |
|  | *aadA22* | 0 (0%) | 8 (1.5%) | 8 (1.4%) | NS |
|  | *aadA24* | 2 (4.0%) | 17 (3.2%) | 19 (3.3%) | NS |
|  | *ant(2")-Ia* | 1 (2.0%) | 8 (1.5%) | 9 (1.6%) | NS |
|  | *ant(3")-Ia* | 24 (48.0%) | 330 (62.6%) | 354 (61.4%) | NS |
|  | *ant(3")-Ii/aac(6')-IId* | 0 (0%) | 2 (0.4%) | 2 (0.3%) | - |
|  | *aph(3")-Ib* | 22 (44.0%) | 205 (38.9%) | 227 (39.3%) | NS |
|  | *aph(3')-Ia* | 6 (12.0%) | 50 (9.5%) | 56 (9.7%) | NS |
|  | *aph(3')-IIa* | 0 (0%) | 1 (0.2%) | 1 (0.2%) | - |
|  | *aph(4)-a* | 0 (0%) | 2 (0.4%) | 2 (0.3%) | - |
|  | *aph(6)-Id* | 22 (44.0%) | 210 (39.8%) | 232 (40.2%) | NS |
|  | *rmtB* | 0 (0%) | 1 (0.2%) | 1 (0.2%) | - |
|  | *rmtE* | 0 (0%) | 1 (0.2%) | 1 (0.2%) | - |
| **Polymyxins** | *mcr* | 0 (0%) | 4 (0.8%) | 4 (0.7%) | - |
|  | *pmrA* mutation R81S | 0 (0%) | 1 (0.2%) | 1 (0.2%) | - |
| **Tetracyclines** | *tetA* | 15 (30.0%) | 276 (52.4%) | 291 (50.4%) | NS |
|  | *tetB* | 15 (30.0%) | 85 (16.1%) | 100 (17.3%) | NS |
|  | *tetC* | 0 (0%) | 2 (0.4%) | 2 (0.3%) | - |
|  | *tetD* | 0 (0%) | 3 (0.6%) | 3 (0.5%) | - |
|  | *tetW* | 0 (0%) | 1 (0.2%) | 1 (0.2%) | - |
|  | 16S rRNA *rrsB* mutation G1058C | 0 (0%) | 1 (0.2%) | 1 (0.2%) | - |
| **Macrolides** | *mefB* | 2 (4.0%) | 27 (5.1%) | 29 (5.0%) | NS |
|  | *mefC* | 0 (0%) | 1 (0.2%) | 1 (0.2%) | - |
|  | *mphA* | 13 (26.0%) | 263 (49.9%) | 276 (47.8%) | NS |
|  | *mphG* | 0 (0%) | 1 (0.2%) | 1 (0.2%) | - |
|  | *ereA* | 0 (0%) | 1 (0.2%) | 1 (0.2%) | - |
|  | *ermB* | 1 (2.0%) | 29 (5.5%) | 30 (5.2%) | NS |
| **Florfenicol** | *floR* | 3 (6.0%) | 40 (7.6%) | 43 (7.5%) | NS |
| **Cephalosporins** | Chromosomal *ampC* promoter mutation,  T-32A | 1 (2.0%) | 0 (0%) | 1 (0.2%) | - |
|  | Chromosomal *ampC* promoter mutation,  C-42T | 4 (8.0%) | 0 (0%) | 4 (0.7%) | - |
