# Supplementary material for: Genetic Predictive Factors for Nonsusceptible Phenotypes and Multidrug Resistance in Expanded-Spectrum Cephalosporin-Resistant Uropathogenic Escherichia coli from a Multicenter Cohort: Insights into the Phenotypic and Genetic Basis of Coresistance
Source: mSphere. 2022 Nov 15;7(6):e00471-22. doi: 10.1128/msphere.00471-22 (PMC9769571; doi:10.1128/msphere.00471-22)
Supplement: TABLE S6 [file msphere.00471-22-s0006.docx]

**Supplementary Table S6:** Plasmid replicon types identified from WGS analysis, stratified by ESBL phenotype. Statistical analyses were performed using Fischer’s exact test in R 3.0.1. In the table, the ‘-’ symbol denotes that the sample size was not sufficient to generate a *p* value, whereas ‘NS’ indicates a non-significant result.

|  | | | | |
| --- | --- | --- | --- | --- |
| **Replicon type** | **Non-ESBL (N=50)** | **ESBL (N=527)** | **Overall (N=577)** | ***p*** |
| Col | 27 (54.0%) | 362 (68.7%) | 389 (67.4%) | NS |
| IncA/C | 2 (4.0%) | 1 (0.2%) | 3 (0.5%) | - |
| IncB/O | 3 (6.0%) | 35 (6.6%) | 38 (6.6%) | NS |
| IncFIA | 19 (38.0%) | 323 (61.3%) | 342 (59.3%) | **0.041** |
| IncFIB | 34 (68.0%) | 401 (76.1%) | 435 (75.4%) | NS |
| IncFIC | 12 (24.0%) | 157 (29.8%) | 169 (29.3%) | NS |
| IncFII | 28 (56.0%) | 382 (72.5%) | 410 (71.1%) | NS |
| IncHI | 17 (34.0%) | 57 (10.8%) | 74 (12.8%) | **<0.001** |
| IncI | 3 (6.0%) | 14 (2.7%) | 17 (2.9%) | NS |
| IncL/M | 0 (0%) | 1 (0.2%) | 1 (0.2%) | - |
| IncN | 3 (6.0%) | 18 (3.4%) | 21 (3.6%) | NS |
| IncP | 0 (0%) | 1 (0.2%) | 1 (0.2%) | - |
| IncQ | 8 (16.0%) | 40 (7.6%) | 48 (8.3%) | NS |
| IncR | 1 (2.0%) | 3 (0.6%) | 4 (0.7%) | - |
| IncX | 8 (16.0%) | 39 (7.4%) | 47 (8.1%) | NS |
| IncY | 4 (8.0%) | 31 (5.9%) | 35 (6.1%) | NS |
