# Supplementary material for: Genetic Predictive Factors for Nonsusceptible Phenotypes and Multidrug Resistance in Expanded-Spectrum Cephalosporin-Resistant Uropathogenic Escherichia coli from a Multicenter Cohort: Insights into the Phenotypic and Genetic Basis of Coresistance
Source: mSphere. 2022 Nov 15;7(6):e00471-22. doi: 10.1128/msphere.00471-22 (PMC9769571; doi:10.1128/msphere.00471-22)
Supplement: TABLE S7 [file msphere.00471-22-s0007.docx]

**Supplementary Table S7:** Matrix displaying positive predictive values (PPVs) for resistance genes of interest vs. antimicrobial non-susceptibility. Isolates which displayed intermediate or resistant susceptibility in relation to CLSI breakpoints were grouped and described as ‘non-susceptible’ for this analysis. Abbreviated drug names correspond to: Amp-Sul = ampicillin/sulbactam, Pip-Taz = piperacillin/tazobactam, TMP-SMZ = trimethoprim/sulfamethoxazole.

|  | **Antimicrobial agent** | | | | | | | | | | |
| --- | --- | --- | --- | --- | --- | --- | --- | --- | --- | --- | --- |
| **Gene** | Amp-Sul | Amikacin | Cefepime | Ciprofloxacin | Ertapenem | Gentamicin | Levofloxacin | Nitrofurantoin | Pip-Taz | Tobramycin | TMP-SMZ |
| CTX-M-14 | 0.773 | 0.000 | 0.364 | 0.750 | 0.023 | 0.227 | 0.864 | 0.227 | 0.023 | 0.205 | 0.523 |
| CTX-M-15 | 0.812 | 0.052 | 0.593 | 0.861 | 0.003 | 0.435 | 0.880 | 0.133 | 0.080 | 0.620 | 0.608 |
| CTX-M-27 | 0.281 | 0.000 | 0.354 | 0.740 | 0.000 | 0.156 | 0.760 | 0.094 | 0.010 | 0.167 | 0.698 |
| CTX-M-55 | 0.646 | 0.000 | 0.479 | 0.792 | 0.000 | 0.542 | 0.813 | 0.125 | 0.063 | 0.500 | 0.646 |
| CMY class | 0.957 | 0.022 | 0.152 | 0.478 | 0.000 | 0.174 | 0.478 | 0.239 | 0.196 | 0.283 | 0.413 |
| DHA class | 1.000 | 0.000 | 0.250 | 0.500 | 0.000 | 0.250 | 0.500 | 0.250 | 0.250 | 0.500 | 1.000 |
| OXA class | 0.962 | 0.082 | 0.598 | 0.973 | 0.005 | 0.614 | 0.973 | 0.158 | 0.136 | 0.962 | 0.630 |
| TEM class | 0.855 | 0.040 | 0.455 | 0.700 | 0.015 | 0.420 | 0.735 | 0.130 | 0.080 | 0.470 | 0.730 |
| *sul* | 0.706 | 0.039 | 0.475 | 0.810 | 0.008 | 0.382 | 0.831 | 0.156 | 0.086 | 0.519 | 0.886 |
| *dfrA/B* | 0.720 | 0.036 | 0.459 | 0.810 | 0.008 | 0.368 | 0.827 | 0.146 | 0.080 | 0.505 | 0.945 |
| *oqx* | 0.500 | 0.000 | 1.000 | 1.000 | 0.000 | 0.500 | 1.000 | 0.000 | 0.500 | 0.500 | 1.000 |
| *qnrB* | 0.769 | 0.000 | 0.308 | 0.615 | 0.000 | 0.538 | 0.538 | 0.000 | 0.000 | 0.692 | 0.769 |
| *qnrS* | 0.618 | 0.000 | 0.324 | 0.588 | 0.000 | 0.176 | 0.676 | 0.059 | 0.029 | 0.265 | 0.706 |
| *aac(6')-Ib-cr* | 0.968 | 0.081 | 0.597 | 0.984 | 0.000 | 0.618 | 0.984 | 0.151 | 0.129 | 0.978 | 0.634 |
| *aac* | 0.901 | 0.034 | 0.552 | 0.916 | 0.005 | 0.970 | 0.916 | 0.167 | 0.079 | 0.916 | 0.640 |
| *aad* | 0.709 | 0.036 | 0.480 | 0.832 | 0.009 | 0.396 | 0.850 | 0.165 | 0.087 | 0.538 | 0.916 |
| *ant* | 0.729 | 0.037 | 0.475 | 0.845 | 0.008 | 0.407 | 0.856 | 0.158 | 0.090 | 0.545 | 0.924 |
| *aph* | 0.649 | 0.027 | 0.429 | 0.764 | 0.000 | 0.371 | 0.784 | 0.120 | 0.054 | 0.425 | 0.838 |
