# Supplementary material for: Genetic Predictive Factors for Nonsusceptible Phenotypes and Multidrug Resistance in Expanded-Spectrum Cephalosporin-Resistant Uropathogenic Escherichia coli from a Multicenter Cohort: Insights into the Phenotypic and Genetic Basis of Coresistance
Source: mSphere. 2022 Nov 15;7(6):e00471-22. doi: 10.1128/msphere.00471-22 (PMC9769571; doi:10.1128/msphere.00471-22)
Supplement: TABLE S8 [file msphere.00471-22-s0008.docx]

**Supplementary Table S8:** Logistic regression analysis to assess the presence of common resistance genes as predictors of antibiotic non-susceptibility and MDR in ESCR UPEC. Generalized linear model using a logit link function and the glm(family = binomial) function in R. Outcomes are binary (1 or 0), with 1 denoting non-susceptibility or MDR status (defined as resistant to at least 1 agent in ≥3 classes of antimicrobial agents). The most common β-lactamase genes, the MLST, ST131, and the acetyltransferase gene, *aac(6’)-Ib-cr*, were included in the analysis. Abbreviated drug names correspond to: Pip-Taz = piperacillin/tazobactam, Amp-Sul = ampicillin/sulbactam, FQ = fluoroquinolones, TMP-SMZ = trimethoprim/sulfamethoxazole, NIT = nitrofurantoin.

|  | **Pip-Tazo** | | **Amp-Sul** | | **FQ** | | **TMP-SMZ** | | **Gentamicin** | | **Amikacin** | | **Tobramycin** | | **NIT** | | **Cefepime** | | **MDR** | |  |
| --- | --- | --- | --- | --- | --- | --- | --- | --- | --- | --- | --- | --- | --- | --- | --- | --- | --- | --- | --- | --- | --- |
|  |  |  |  |  |  |  |  |  |  |  |  |  |  |  |  |  |  |  |  |  |  |
| **Predictor** | OR  (95% CI) | *p* | OR  (95% CI) | *p* | OR  (95% CI) | *p* | OR  (95% CI) | *p* | OR  (95% CI) | *p* | OR  (95% CI) | *p* | OR  (95% CI) | *p* | OR  (95% CI) | *p* | OR  (95% CI) | *p* | OR  (95% CI) | *p* |  |
| **ST131** | 0.4 (0.3 - 1.5) | 0.282 | 1.1 (0.7 - 2.0) | 0.63 | 5.7  (3.0 - 12.0) | **<0.001** | 0.8  (0.5 - 1.2) | 0.3 | 1.5 (1.0 - 2.4) | 0.06 | 1.4 (0.5 - 4.6) | 0.609 | 1.4  (0.7 - 2.5) | 0.322 | 0.8 (0.4 - 1.3) | 0.339 | 1.8 (1.2 - 2.6) | **0.005** | 2.2  (1.4 - 3.7) | **0.001** | |
|  |  |  |  |  |  |  |  |  |  |  |  |  |  |  |  |  |  |  |  |  | |
| **CTX-M-14** | 0.5 (0.02 - 4.24) | 0.589 | 1.9 (0.7 - 5.9) | 0.243 | 7.8  (2.6 - 26.0) | **<0.001** | 0.6  (0.2 - 1.4) | 0.228 | 0.7 (0.3 - 2.0) | 0.534 | 0.0  (-) | 0.995 | 0.7  (0.2 - 2.1) | 0.479 | 3.1 (0.9 - 10.7) | 0.071 | 2.5 (0.9 - 6.9) | 0.073 | 1.3  (0.5 - 3.4) | 0.55 | |
|  |  |  |  |  |  |  |  |  |  |  |  |  |  |  |  |  |  |  |  |  | |
| **CTX-M-15** | 0.7 (0.2 - 2.8) | 0.564 | 0.8 (0.3 -1.8) | 0.545 | 3.3  (1.5 - 7.5) | **0.004** | 0.8  (0.4 - 1.6) | 0.483 | 0.6 (0.3 - 1.4) | 0.267 | 0.9 (0.14 - 10.6) | 0.885 | 0.5  (0.2 - 1.4) | 0.186 | 1.3 (0.5 - 3.9) | 0.655 | 5.2 (2.4 - 12.4) | **<0.001** | 0.6  (0.3 - 1.2) | 0.145 | |
|  |  |  |  |  |  |  |  |  |  |  |  |  |  |  |  |  |  |  |  |  | |
| **CTX-M-27** | 0.3 (0.1 - 2.2) | 0.285 | 0.2 (0.1 - 0.6) | 0.002 | 2.1  (0.9 - 5.0) | 0.096 | 1.7  (0.8 - 3.9) | 0.176 | 0.5 (0.2 - 1.4) | 0.191 | 0.0  (-) | 0.993 | 0.7  (0.3 - 2.0) | 0.538 | 1.1 (0.3 - 3.6) | 0.93 | 2.0 (0.8 - 4.9) | 0.142 | 1.4  (0.6 - 3.2) | 0.405 | |
|  |  |  |  |  |  |  |  |  |  |  |  |  |  |  |  |  |  |  |  |  | |
| **CTX-M-55** | 1.0 (0.2 - 4.6) | 0.958 | 0.9 (0.3 - 2.5) | 0.802 | 6.3  (2.4 - 17.4) | **<0.001** | 1.2  (0.5 - 3.0) | 0.631 | 4.5 (1.8 - 11.6) | **0.002** | 0.0  (-) | 0.995 | 4.2  (1.5 - 11.9) | **0.006** | 1.2 (0.3 - 3.8) | 0.794 | 4.7 (1.9 - 12.4) | 0.001 | 3.7  (1.5 - 9.4) | **0.004** | |
|  |  |  |  |  |  |  |  |  |  |  |  |  |  |  |  |  |  |  |  |  | |
| **CMY-2** | 4.9 (1.3 - 20.4) | **0.02** | 33.4 (4.0 - 626.1) | 0.001 | 0.6  (0.3 - 1.7) | 0.355 | 0.2 (0.1 - 0.6) | 0.002 | 0.4 (0.1 - 1.1) | 0.072 | 1.4 (0.1 - 20.1) | 0.826 | 0.6  (0.2 - 1.9) | 0.413 | 2.6 (0.9 - 8.2) | 0.09 | 0.5 (0.1 - 1.4) | 0.195 | 0.5  (0.2 - 1.2) | 0.106 | |
|  |  |  |  |  |  |  |  |  |  |  |  |  |  |  |  |  |  |  |  |  | |
| **TEM-1B** | 1.49 (0.7 - 3.14) | 0.304 | 10.3 (5.8 - 19.3) | <0.001 | 0.8  (0.5 - 1.28) | 0.3 | 2.7 (1.8 -4.1) | <0.001 | 3.4 (2.2 - 5.5) | **<0.001** | 1.8 (0.6 - 5.2) | 0.268 | 5.4  (3.2 - 9.6) | **<0.001** | 0.8 (0.4-1.4) | 0.42 | 1.1 (0.7 - 1.5) | 0.922 | 2.0  (1.3 - 3.5) | **0.002** | |
|  |  |  |  |  |  |  |  |  |  |  |  |  |  |  |  |  |  |  |  |  | |
| **OXA-1** | 4.8 (0.5 - 53.4) | 0.223 | 2.3 (0.3 - 13.3) | 0.382 | 0.7  (0.1 - 7.6) | 0.727 | 0.6 (0.2 - 2.1) | 0.474 | 2.7 (0.8 - 9.0) | 0.104 | 4.0 (0.3 - 83.1) | 0.399 | 8.6  (1.5 - 43.2) | **0.01** | 4.5 (0.8 - 28.9) | 0.107 | 1.0 (0.3 - 3.2) | 0.988 | 2.9  (0.4 - 20.5) | 0.285 | |
|  |  |  |  |  |  |  |  |  |  |  |  |  |  |  |  |  |  |  |  |  | |
| ***aac(6’)-Ib-cr*** | 1.5 (0.1 - 14.1) | 0.771 | 15.2 (2.7 - 121.5) | 0.005 | 28.7 (2.4 - 418.7) | **0.01** | 2.7 (0.8 - 10.9) | 0.127 | 3.0 (0.9 - 10.4) | 0.08 | 1.4 (0.1- 23.4) | 0.844 | 58.1  (12.1 - 387.2) | **<0.001** | 0.4 (0.1 - 2.2) | 0.281 | 1.0 (0.3 - 3.4) | 0.979 | 33.8 (4.9 - 313) | **0.001** | |
|  |  |  |  |  |  |  |  |  |  |  |  |  |  |  |  |  |  |  |  |  | |
