# Supplementary material for: Genetic Predictive Factors for Nonsusceptible Phenotypes and Multidrug Resistance in Expanded-Spectrum Cephalosporin-Resistant Uropathogenic Escherichia coli from a Multicenter Cohort: Insights into the Phenotypic and Genetic Basis of Coresistance
Source: mSphere. 2022 Nov 15;7(6):e00471-22. doi: 10.1128/msphere.00471-22 (PMC9769571; doi:10.1128/msphere.00471-22)
Supplement: TABLE S9 [file msphere.00471-22-s0009.docx]

**Supplementary Table S9:** Logistic regression analysis to assess the presence of common resistance genes as predictors of antibiotic non-susceptibility to fluoroquinolones in ESCR UPEC. Generalized linear model used a logit link function and the glm(family = binomial) function in R. Outcomes are binary (1 or 0).

| **Predictors** | **Odds Ratios** | **95% Confidence Interval** | ***p*** |
| --- | --- | --- | --- |
| ST131 | 3.06 | (1.49 – 6.61) | **0.003** |
| CTX-M-14 | 3.73 | (0.98 – 15.67) | 0.061 |
| CTX-M-15 | 2.64 | (0.98 – 7.19) | 0.055 |
| CTX-M-27 | 1.07 | (0.37 – 3.10) | 0.896 |
| CTX-M-55 | 2.92 | (0.93 – 9.88) | 0.073 |
| CMY-2 | 0.61 | (0.19 – 2.00) | 0.410 |
| TEM-1B | 0.36 | (0.19 – 0.66) | **0.001** |
| OXA-1 | 0.23 | (0.03 – 2.90) | 0.204 |
| *aac(6’)-Ib-cr* | 56.48 | (4.08 – 865.12) | **0.003** |
| gyrase and/or topoisomerase IV mutations | 16.42 | (8.84 – 31.77) | **<0.001** |
